# Monitoring Polymorphic Phase Transitions in Flufenamic Acid Amorphous Solid Dispersions Using Hyphenated X-ray Diffraction–Differential Scanning Calorimetry

**DOI:** 10.1021/acs.molpharmaceut.2c00016

**Published:** 2022-03-29

**Authors:** Yuying Pang, Asma Buanz, Simon Gaisford, Oxana V. Magdysyuk, Gareth R. Williams

**Affiliations:** † UCL School of Pharmacy, University College London, 29-39 Brunswick Square, London WC1N 1AX, United Kingdom; ‡ Diamond Light Source, Harwell Science and innovation Campus, Didcot, Oxfordshire OX11 0DE, United Kingdom

**Keywords:** amorphous solid dispersion, hydroxypropylmethylcellulose, ethyl cellulose, flufenamic acid, X-ray diffraction, differential scanning calorimetry, polymorphic transition

## Abstract

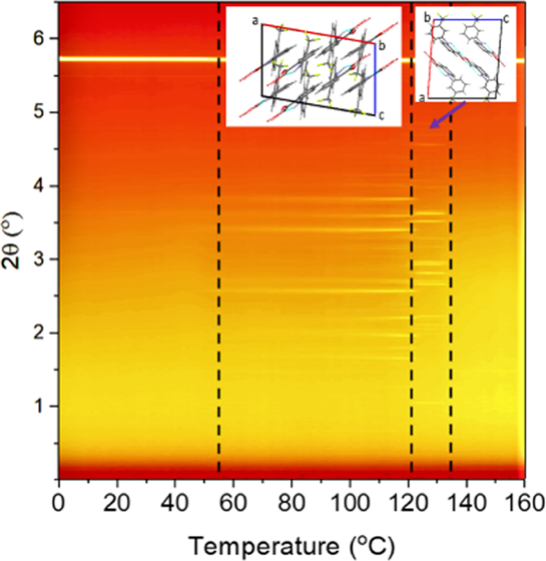

Flufenamic acid (FFA) is a highly polymorphic drug molecule with nine crystal structures reported in the Cambridge Structural Database. This study explores the use of synchrotron X-ray powder diffraction combined with differential scanning calorimetry to study crystallization and polymorphic phase transitions upon heating FFA–polymer amorphous solid dispersions (ASDs). Ethyl cellulose (EC, 4 cp) and hydroxypropylmethylcellulose (HPMC) grades with different viscosities and substitution patterns were used to prepare dispersions with FFA at 5:1, 2:1, 1:1, and 1:5 w/w drug/polymer ratios by quench cooling. We employed a 6 cp HPMC 2910 material and two HPMC 2208 samples at 4000 and 100 000 cp. Hyphenated X-ray diffraction (XRD)–differential scanning calorimetry (DSC) studies show that the 6 and 100 000 cp HPMCs and 4 cp EC polymers can stabilize FFA form IV by inhibiting the transition to form I during heating. It appears that the polymers stabilize FFA in both amorphous and metastable forms via a combination of intermolecular interactions and viscosity effects. Increasing the polymer content of the ASD also inhibits polymorphic transitions, with drug/polymer ratios of 1:5 w/w resulting in FFA remaining amorphous during heating. The comparison of FFA ASDs prepared with different samples of HPMCs and ECs suggests that the chemical substitution of the polymer (HPMC 2208 has 19–24% methoxy groups and 4–12% hydroxypropyl groups, while HPMC 2910 has 28–30% methoxy groups and 7–12% hydroxypropyl groups) plays a more significant role in directing polymorphic transitions than the viscosity. A previously unreported polymorph of FFA was also noted during heating but its structure could not be determined.

## Introduction

For many poorly soluble active pharmaceutical ingredients (APIs), the use of the amorphous form is a widely explored route to improve bioavailability. The major problem with this is that the amorphous form is thermodynamically unstable and will spontaneously crystallize to more stable crystalline counterparts upon storage. Crystallization is a complicated process during which a number of polymorphs may coexist within the same formulation.^1−3^ Polymeric additives are often applied to increase the stability of amorphous materials by inhibiting crystallization.^4,5^ Several studies have discussed the specific interactions (such as hydrogen bonding) and steric considerations (owing to the viscous nature of polymers), which play a role in delaying crystallization and inhibiting crystal growth, but the processes are still not well understood.^3,4,6^

Flufenamic acid (FFA; 2-[3-(trifluoromethyl)amino]benzoic acid), also known as fenamate, is a nonsteroidal anti-inflammatory drug (NSAID) used for treating rheumatoid arthritis, osteoarthritis, and various musculoskeletal pain conditions.^7^ It belongs to class II of the Biopharmaceutical Classification System (BCS), with low solubility and high permeability.^8^ This results in low bioavailability in vivo and hence to suboptimal therapeutic outcomes. To overcome this limitation, amorphization has been explored to improve the solubility of FFA.^9^

**Figure 1:**
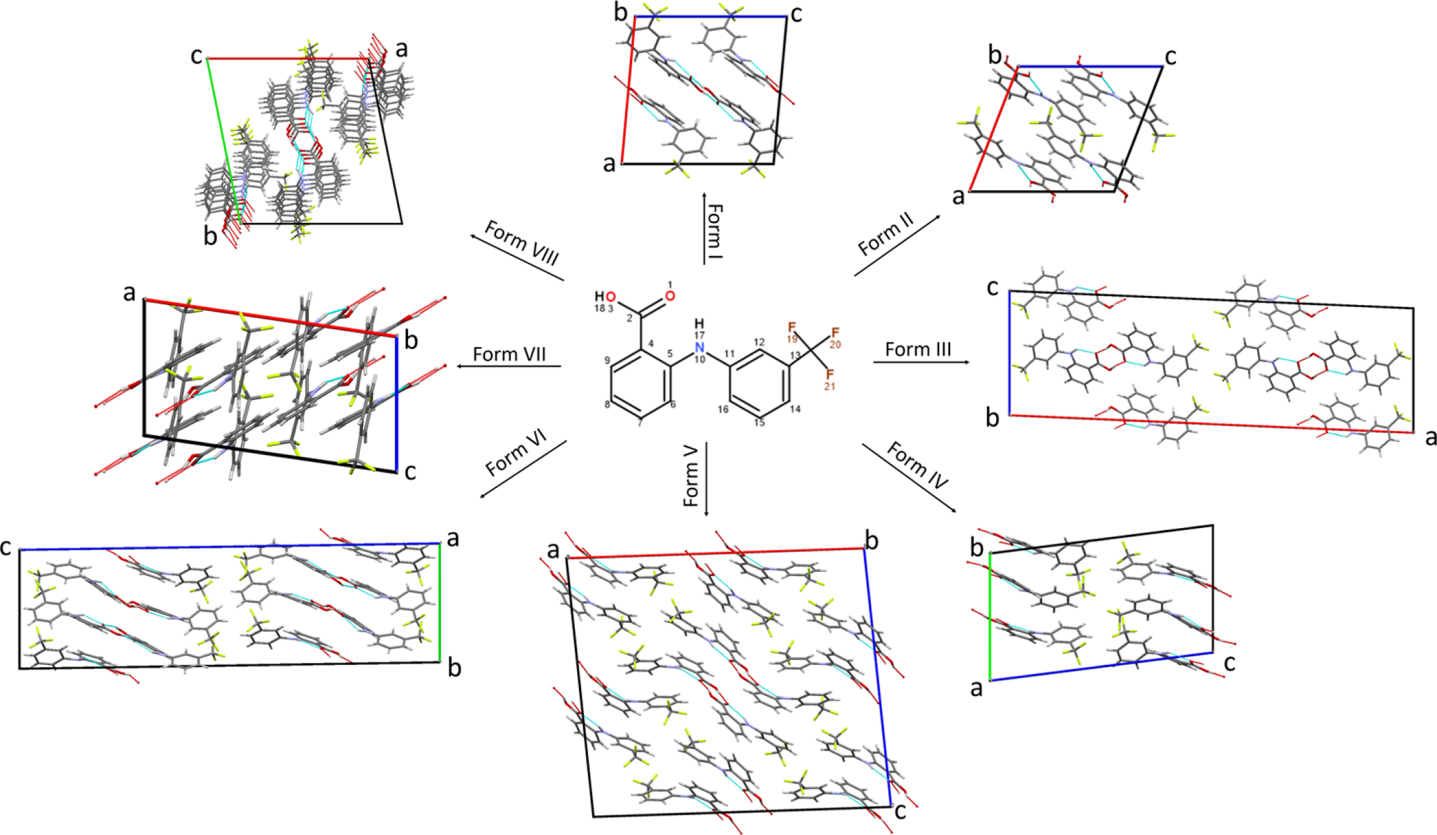
Molecular packing of FFA form I to form VIII. Forms I (CSD reference: FPAMCA11), II (FPAMCA17), III (FPAMCA), V (FPAMCA16), and VII (FPAMCA12) viewed down the *b*-axis, form IV (FPAMCA15) and VI (FPAMCA14) viewed down the *a*-axis, and form VIII viewed down the *c*-axis; disorder present in the structure of form IV is omitted for clarity.^10,12^

The polymorphism of FFA has been the subject of extensive research by both the crystallographic and pharmaceutical communities for more than 40 years. According to the Cambridge Structural Database (CSD), nine polymorphs of FFA have been discovered (search performed on September 30, 2021), including eight structurally characterized forms (Figure 1). The crystallographic data are listed in the Supporting Information, Table S1.^10^ The first crystal structure of FFA (form III) was elucidated in 1973, and nearly 10 years later, form I was reported.^11,12^ Form II and forms IV–VIII were reported more recently in 2011.^12^ The precise order of stabilities of the FFA polymorphs and their transitions is not well understood despite extensive research efforts. In all eight characterized FFA systems, a strong intramolecular O···H–N hydrogen bond holds the phenyl ring carboxylic group and bridging amino group coplanar. While packing, FFA molecules form dimers and the dominant intermolecular forces in the structures are O···H–O hydrogen bonds between adjacent molecules.^12^

Differential scanning calorimetry (DSC) is a powerful tool that has been widely used to investigate the phase transitions of APIs, both alone and in combination with excipients. For instance, DSC has been shown to be very powerful in exploring crystallization from drug/polymer amorphous solid dispersions (ASDs) upon heating.^13^ However, structural information cannot be obtained from DSC profiles; to acquire this, X-ray diffraction (XRD) is required. Because of the fast heating rates used in DSC, XRD and DSC measurements are generally performed separately. Hyphenating the techniques to obtain DSC and XRD data simultaneously on the same sample has been shown to add significant additional insight. Recently, the combination of high-energy synchrotron XRD with differential scanning calorimetry (XRD–DSC) has been applied to study phase transitions in a range of systems including carbamazepine,^14^ mefenamic acid,^15^ paracetamol/lactose blends,^16^ and spray-dried ASDs of olanzapine.^13^

In this paper, ASDs of FFA–polymer were prepared, and the heat-induced crystallization of FFA from the dispersion was explored by XRD–DSC. The polymorphic phase transitions observed were investigated in detail. Ethyl cellulose (EC, 4 cp) and grades of hydroxypropylmethylcellulose (HPMC) with different viscosities (6, 4000, and 100 000 cp) were used as the polymer additives. HPMCs are a family of soluble methylcellulose ethers. They are hydrophilic, biodegradable, and biocompatible polymers having a wide range of applications in drug delivery. HPMC polymers are available in various viscosity grades ranging from 3 to 100 000 cp.^17,18^ Here, HPMC was used to investigate how polymer viscosity and chemical structure affect the polymorphic transitions of FFA. EC is a hydrophobic cellulose ether used as a coating material, a tablet binder, and a matrix former, with viscosity from 4 to 300 cp.^19^ FFA/EC dispersions were prepared and compared with FFA/HPMC systems to explore how the hydrophobicity of the carrier matrix affects the polymorphic transitions upon crystallization.

## Experimental Methods

### Materials

Flufenamic acid (C_14_H_10_F_3_NO_2_; *M*_w_ = 281.23 g/mol) was purchased from Acros. HPMC 2208 with a viscosity of 4000 cp (K4M) and 100 000 cp (K100M) was purchased from Colorcon Ltd., and HPMC 2910 (PharmaCoat 606; viscosity: 6 cp) from ShinEtsu. EC (4 cp) was obtained from Sigma-Aldrich.

### Simultaneous XRD–DSC Analysis

The experimental setup for XRD–DSC is very similar to that described in our previous work.^15^ XRD–DSC experiments were carried out on the Joint Engineering, Environment and Processing Beamline I12 (JEEP) at the Diamond Light Source.^20^ A modified Q20 DSC (TA Instruments) was mounted and aligned on the sample stage in the experimental hutch to allow the monochromated X-ray beam (0.5 mm × 0.5 mm; λ = 0.234 Å) pass through holes in the DSC cell. A Thales Pixium RF4343 detector was located 1.9 m behind the sample. The DSC was calibrated with an indium standard and the Pixium detector with cerium dioxide, prior to experiments beginning. Initial experiments were performed with FFA alone. Form I was heated from 0 to 150 °C and then equilibrated to 0 °C. The same sample was then heated to 70 °C at 10 °C/min and held at 70 °C for 5 min.

To prepare ASDs, appropriate amounts of FFA, HPMC, and EC were weighed in 5 mL glass vials to give a final mass of ca. 30 mg and mass ratios of 5:1, 2:1, 1:1, and 1:5 (w/w FFA/HPMC; FFA/EC). The samples were then mixed for ca. 3 min using a vortex mixer. Approximately 20 mg of each sample was heated in the DSC from 0 to 150 °C at 10 °C/min before equilibrating back to 0 °C to produce ASDs. Finally, the products were heated to 160 °C at 10 °C/min. The FFA/HPMC (4000 cp) 1:1 ASDs were also ramped from 0 to 160 °C at 2 °C/min in a separate experiment.

The Diamond Generic Data Acquisition (GDA) software was employed to collect diffraction patterns for 5 s, with a 1 s pause between each scan, which equated to a pattern for every 1 °C of heating. The two-dimensional (2D) Pixium data sets were masked and converted into one-dimensional (1D) diffraction patterns by azimuthal integration using the DAWN Science Workbench.^21−23^ TOPAS-Academic V5^24^ was employed to analyze selected patterns with the Rietveld method^25,26^ implemented within the software to obtain realistic values for the unit cell parameters at elevated temperatures.^27^ Structural data for the FFA polymorphs were obtained from the CSD. Batch Rietveld refinements were then performed to determine phase fractions as a function of temperature.

### Fourier Transform Infrared Spectroscopy (FTIR)

FTIR spectroscopy was conducted on a Perkin Elmer Spectrum 100 instrument. All spectra were recorded between 650 and 4000 cm^–1^ with 64 scans at a resolution of 4 cm^–1^. FFA, HMPC, EC, and the samples prepared for the DSC–XRD study were all explored. The drug/polymer mixtures were heated in the DSC from 0 to 150 °C at 10 °C/min before equilibrating back to 0 °C. Finally, the products were heated to the temperature after each recrystallization event at 10 °C/min. The samples were then removed from the DSC and characterized by FTIR.

### Laboratory XRD

Standard laboratory XRD experiments were undertaken on a MiniFlex 600 diffractometer (Rigaku) supplied with Cu Kα radiation (λ = 0.15  418 nm, 40 kV, 15 mA). FFA was loaded in low-volume glass holders and scanned from 5 to 50° in 0.02° steps at 2°/min. The experimental data were plotted with OriginPro 2017 and compared with calculated patterns for the various polymorphic forms obtained from the CSD.

### Variable-Temperature Laboratory XRD (VT-XRD)

VT-XRD measurements were performed using a Stoe Stadi-P diffractometer equipped with a Cu anode (Kα1), a Ge monochromator, a Dectris Mythen 1K detector, and an Oxford Instruments CryojetHT (90–500 K). An in-house setup was employed to discourage the formation of ice on the goniometer head at low temperatures. FFA raw material was first ground for approx. 10 min and then loaded in a 0.5 mm glass capillary. In one experiment, the FFA-loaded capillary was heated to 145 °C and then quenched to 0 °C before being heated from 30 to 80 °C in 10 °C steps. In the second run, ice water was used to quench the sample, but all other parameters were kept the same. XRD patterns were obtained on reheating from 30 to 80 °C. The sample was scanned from 2 to 50° 2θ in steps of 0.5° at 10 s per step. A complete scan lasted approx. 23 min, and each 10 °C temperature change took approx. 12 min. Once reaching the desired temperature, the sample was kept at the set temperature for 5 min before starting the next scan.

## Results

### FFA Alone

FFA form I was heated from 0 to 150 °C, equilibrated to 0 °C, and then heated up to 160 °C. During reheating, pure FFA experiences three different transitions and finally recrystallizes into form I (Supporting Information, Figures S1 and S2) before melting. The sample first recrystallizes at 54 °C and then converts to form IV at 74 °C before transforming into form I at 97 °C. This then melts at 133 °C.

**Figure 2:**
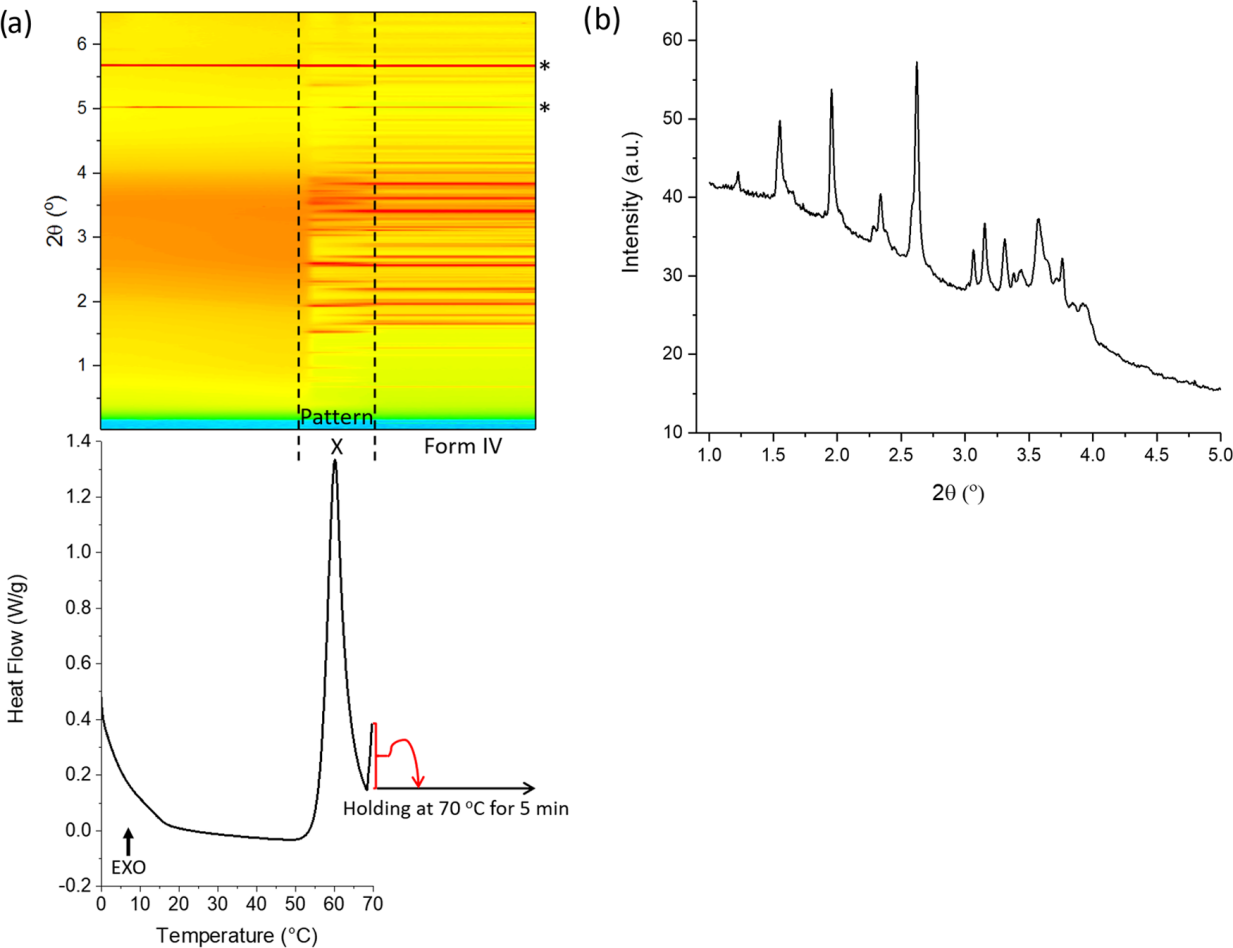
(a) DSC–XRD data obtained on heating a quench-cooled sample of pure FFA to 70 °C and then holding isothermally at this temperature (reflections marked with asterisks are a result of the aluminum pan). (b) The XRD pattern (“X”) obtained by cooling melted FFA to 0 °C and then reheating to 70 °C. Data in panel (a) are plotted as a function of temperature, with the *x*-axis in both the top and bottom panels covering the same temperature range. XRD data (λ = 0.234 Å) at the top of panel (a) are plotted as a heat map, with darker red colors indicating greater diffracted intensity.

The crystallinity of the material present at 70 °C upon heating the FFA glass is poor, but the pattern cannot be satisfactorily matched with any polymorphic form of FFA (Figure S3). This new FFA pattern is subsequently termed pattern X (form X). In attempts to obtain form X with better crystallinity, FFA form I was heated from 0 to 150 °C and then equilibrated to 0 °C to produce amorphous FFA. The reheating process was monitored by XRD–DSC, with the sample heated to 70 °C at 10 °C/min and then held at 70 °C for 5 min. Figure 2 shows that reflections appear at ca. 50 °C together with an exothermic peak. Changes in the positions of the Bragg reflections are observed in the XRD data after holding at 70 °C for 5 min, suggesting that a polymorphic transition occurred during the isothermal period.

The pattern obtained at 68 °C is identical to that in Figure S3 and confirms that FFA recrystallized into form X before the isothermal hold period. The major reflections of FFA form X are summarized in Table S2. Computationally predicted crystal structures were explored in attempts to solve the structure of form X. However, none of them provides a satisfactory fit with pattern X. The closest gives an *R*_wp_ (weight profile *R*-factor) of 18 (Figures S4 and S5 and Table S3).

VT-XRD was employed in attempts to obtain high-resolution data for form X. For a sample cooled by ice water, the amorphous form directly recrystallized into form IV (Figure S6). Cooling using a CryojetHT revealed that the amorphous form had crystallized into form IV at 50 and 60 °C; at 40 °C, the pattern appears to correspond to a mixture of forms IV and X, but the crystallinity of the pattern is too poor to determine the crystal structure (Figure S7). A further attempt to prepare phase-pure form X involved melting FFA in an oven at 145 °C (above the melting point of FFA form I but below the degradation temperature, thus ensuring complete melting but no alteration to the molecular structure) and immediately transferring to a diffractometer (Figure S8), but this resulted in a mixture of forms III and IV FFA. Despite myriad attempts to solve its structure, form X could only be clearly identified when heating an FFA glass in a DSC. This suggests that it is very unstable.

### FFA–6 cp HPMC Dispersions

**Figure 3:**
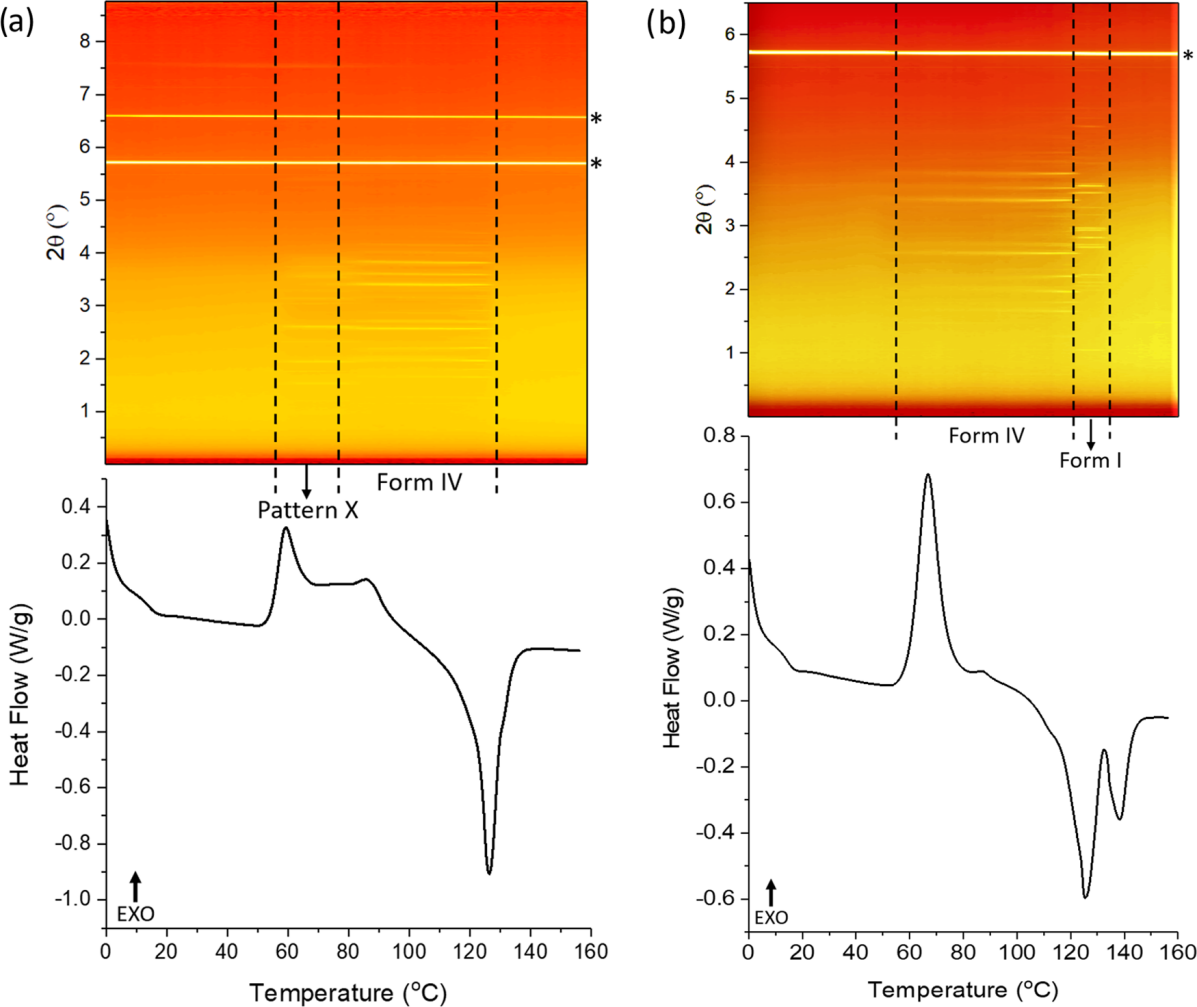
XRD–DSC data obtained when heating a quench-cooled (a) 5:1 w/w FFA/HPMC 6 cp ASD and (b) 5:1 w/w FFA/HPMC 4000 cp ASD. Reflections marked with asterisks arise from the aluminum pan. Data are plotted as a function of temperature, with the *x*-axis in both the top and bottom panels covering the same temperature range. XRD data (λ = 0.234 Å) at the top are plotted as a heat map, with brighter yellow colors indicating greater diffracted intensity.

Combined XRD–DSC data for an FFA/6 cp HPMC 2910 ASD (5:1 w/w) can be seen in Figure 3a.

The XRD–DSC data illustrate the occurrence of an amorphous-to-crystalline transition, the conversion of one polymorph to another, and a crystalline-to-liquid transition. There are no reflections (bar those from the Al pan) in the contour plot until 56 °C when an exothermic event is observed in the DSC trace. A change in the position of the Bragg reflections appears at around 80 °C, with another exothermic peak showing on the DSC thermogram. Finally, at 126 °C, the peak of the endothermic event, a total loss of Bragg reflections in the contour plot is seen.

**Figure 4:**
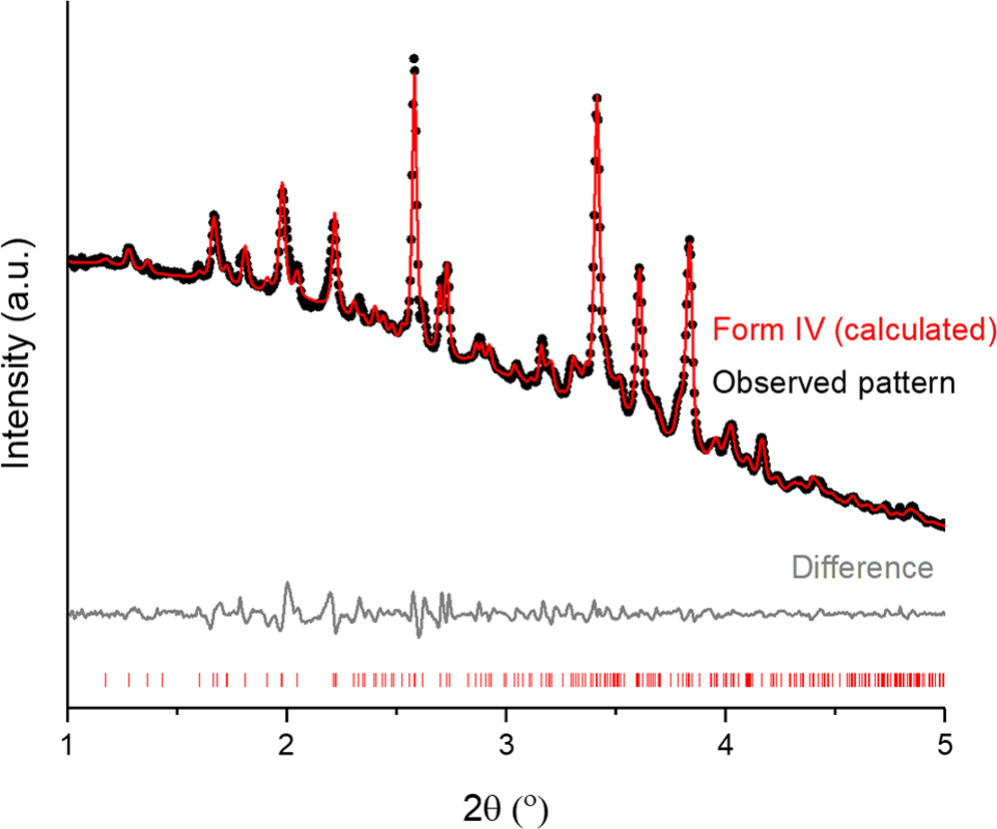
Rietveld refinement on the diffraction pattern recorded for the 5:1 w/w FFA/HPMC 6 cp ASD at 116 °C; tick marks show the position of allowed reflections from FFA form IV (FPAMCA15).

Phase identification was carried out using Rietveld refinement against patterns obtained at 71 and 116 °C. The pattern at 71 °C is the same as pattern X obtained by reheating a pure FFA glass (Figure S9). The pattern recorded at 116 °C shows the FFA to be present as form IV (Figure 4 and Table 1). The refinement thus suggests that the melting endotherm (onset: 121 °C) in Figure 3a is the melting peak of form IV. This is slightly lower than the melting temperature reported by López-Mejías et al. (123 °C),^28^ as would be expected given the presence of the polymer in the composite.

**Table 1:** Refinement Parameters for the 5:1 w/w FFA/HPMC 6 cp ASD at 116 °C^a^

form	IV
temperature (°C)	116
space group	*P*1̅
*a* (Å)	8.7781(16)
*b* (Å)	11.992(1)
*c* (Å)	20.067(3)
α (deg)	80.496(13)
β (deg)	81.087(14)
γ (deg)	74.055(8)
*R*_wp_	1.6701
phase fraction^b^	

a The starting model was taken from the CSD (form IV: FPAMCA15).

b The phase fraction cannot be calculated because there is no reference for pattern X.

Compared with pure FFA, which experienced three different transitions and finally recrystallizes into form I under the same heating conditions (Figure S2), the polymorphic transitions of the FFA/HPMC 6 cp ASD (5:1 w/w) are somewhat different, as the ASD recrystallizes into form X before converting to form IV. This suggests that HPMC inhibits the conversion from form IV to I and stabilizes form IV during heating.

When the FFA/HPMC ratio decreased to 2:1 w/w, XRD–DSC shows that the diffracted intensity in the contour plot is very weak, which may arise from the low crystallinity of the recrystallized material (Figure S10). Very weak Bragg reflections at 1.75, 2, and 2.25° could be observed between 0 and 20 °C, which suggests that the FFA/HPMC (2:1 w/w) mixture was not fully amorphous after quench cooling. However, these reflections are too small to allow the identification of the polymorphic form present. More obvious Bragg reflections can be seen to appear in the contour plot at about 40 °C. These disappear at approx. 120 °C, corresponding to the recrystallization and melting of FFA (Figure S10). Rietveld refinements against the recorded pattern at 86 °C show that FFA recrystallized into form IV (Figure S11 and Table S4). The endothermic peak at 120 °C thus corresponds to the melting of form IV. This is a further depression (cf. the 5:1 w/w ASD) of the literature melting point for FFA IV,^28^ consistent with the presence of additional polymer in the 2:1 system. Batch refinements were then undertaken against all of the patterns, with the *R*_wp_ value ranging from 0.6351 to 5.1667. It can be seen (Figure S12) that FFA form IV begins to grow in at ca. 60 °C, reaches a maximum at around 110 °C, and then declines until no crystalline material remains at 120 °C.

For dispersions prepared with FFA/6 cp HPMC at 1:1 and 1:5 w/w ratios (Figure S13), a small and broad endotherm peaking at approx. 120 °C is observed in the DSC traces, while no exothermic events are visible. For the 1:1 w/w sample, there are some reflections between 80 and 110 °C but they are very weak; no reflections at all can be seen with the 1:5 analogue. The patterns obtained at 100 °C with both samples are shown in Figure S14 and reveal that both are almost entirely amorphous. These systems contain more polymer than the previously discussed 5:1 and 2:1 w/w materials, and at such drug/polymer ratios, it appears that the steric hindrance and viscosity of the polymer provide significant resistance to the movement of drug molecules, severely restricting (1:1 w/w) or preventing (1:5 w/w) the recrystallization of FFA. The low intensity of the peaks present in the XRD patterns makes it hard to identify the polymorph of FFA present, and the broadness of the DSC endotherm also prevents a definitive assignment of which species is melting. However, the reflections seen for the 1:1 system over the temperature range of ca. 75–115 °C appear consistent with FFA form IV.

### FFA–4000 cp HPMC Dispersions

Combined XRD–DSC data for the reheating of an FFA/4000 cp HPMC 2208 dispersion with a 5:1 w/w ratio are presented in Figure 3b. Bragg reflections first appear at 50 °C, corresponding to the onset temperature of the exothermic peak in the DSC trace. A change of the positions of Bragg reflections occurs at about 120 °C, coincident with the onset of an endothermic event in the DSC thermogram. Following this, there is another endothermic peak at 133 °C (melting of form I^28^), at which point a total loss of Bragg reflections is observed. The patterns recorded at 120 and 133 °C were analyzed using the Rietveld method (Figure S15 and Table S5). The pattern recorded at 120 °C reveals FFA to exist as form IV, while at 133 °C, FFA is present as form I.

Dispersions of FFA/4000 cp HPMC at both 2:1 and 1:1 w/w ratios have a similar phase transition behavior (Figure S16). Both samples are in the amorphous form at the beginning of the experiment, and Bragg reflections begin to emerge at about 60 °C. These correspond to FFA form IV, coinciding with the onset of an exothermic peak in the DSC traces. Following this, there is an endothermic event peaking at 126 °C in each thermogram, after which no Bragg reflections can be seen. Finally, in each trace, there is another small endothermic peak with an onset temperature at 134 °C. The diffraction patterns of the two samples do not quite mirror the DSC results. According to the refinements (Figure S17 and Table S6), form IV is the only polymorph that exists in the samples, but there are two endothermic peaks seen in each DSC trace, indicating that there is more than one polymorph in each sample. The onset temperature of the second endothermic peak is 134 °C, the melting temperature of form I. We thus hypothesize that a small amount of form IV converts to form I at this temperature, sufficient to be identified by DSC but not enough to be detectable by XRD.

For an FFA/HPMC 4000 cp ASD at a 1:5 w/w ratio, no obvious reflections can be observed on the counter plot. The two peaks in the DSC trace may relate to the crystallization and then melting of FFA, respectively (Figure S18). However, because of the high viscosity and steric hindrance provided by the HPMC, only a tiny amount of amorphous FFA is recrystallized (Figure S19), barely at the detection limitation of XRD. Therefore, at this drug/polymer ratio, the viscosity of the polymer matrix provides a high level of resistance to the movement of drug molecules, preventing the recrystallization of FFA.

### 1:1 w/w FFA/4000 cp HPMC Dispersion with a 2 °C/min Heating Rate

**Figure 5:**
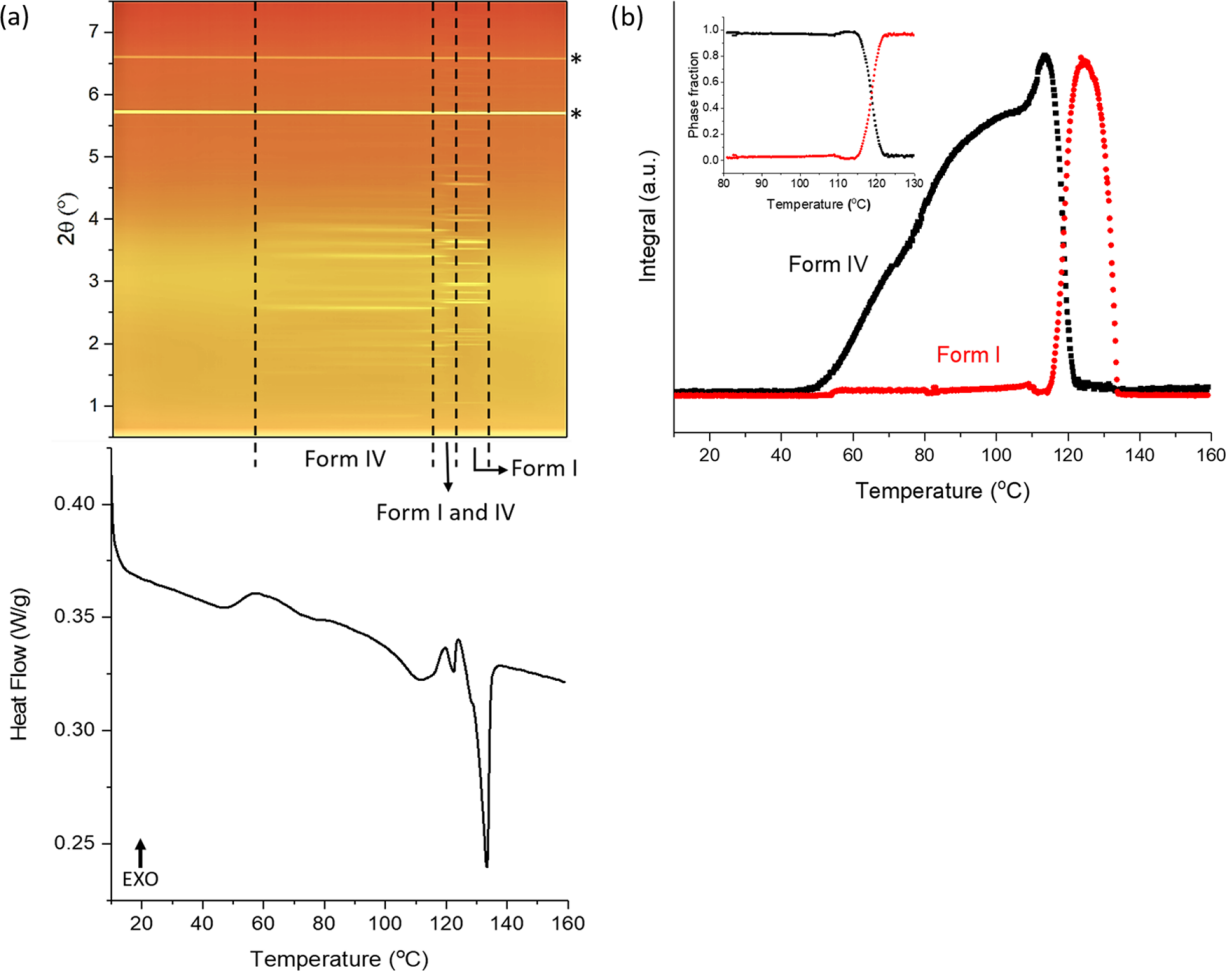
Results of heating the 1:1 w/w FFA/HPMC 4000 cp ASD at 2 °C/min. (a) XRD–DSC data (reflections marked with asterisk are a result of the aluminum pan); (b) plot of integrated total diffracted intensity vs temperature, with inset plot of phase fraction as a function of temperature. Data in panel (a) are plotted as a function of temperature, with the *x*-axis in both the top and bottom panels covering the same temperature range. XRD data (λ = 0.234 Å) at the top are plotted as a heat map, with brighter yellow colors indicating greater diffracted intensity.

The data in the previous section illustrate that there are some discrepancies between the diffraction patterns of the 1:1 and 2:1 w/w FFA/4000 cp HPMC ASDs and the DSC observations. To try and understand this better, the experiment was repeated at a heating rate of 2 °C/min with the 1:1 w/w FFA/4000 cp HPMC dispersion (Figure 5a). The data illustrate that the sample is amorphous at the start of the experiment. At 50 °C, there is a broad exothermic peak in the DSC thermogram, which coincides with the appearance of Bragg reflections of form IV on the XRD contour plot (see also Figure S20 and Table S7). From 113 to 123 °C, the DSC data show a small exotherm–endotherm event, together with a change in the positions of Bragg reflections (form IV + I). Another change in the pattern of Bragg reflections is observed at ca. 123 °C (form I), followed by a total loss of diffracted intensity at about 133 °C. These events are coincident with the peak of the melting endotherms of forms IV and I, respectively.

The integrated total diffraction intensity for each pattern as a function of temperature is plotted in Figure 5b. There is no crystalline material present below 50 °C. The amount of form IV grows with increasing temperature, peaking at 113 °C. After this temperature, the decay of form IV is observed together with the growth of form I. The amount of form IV reaches its lowest point at 120 °C, which means that there is very little form IV left when the second endothermic event occurs.

The plot of phase fractions as a function of temperature (Figure 5b (inset)) reveals the curves of forms I and IV to cross at 0.5, indicating that the polymorph conversion occurred without any wholesale melt. In the DSC profile, the onset temperature of the second endothermic peak is 120 °C, but form I does not start to grow until about 113 °C. The amount of form IV reaches a minimum at 122 °C, its melting point.^12^ These observations suggest that the polymorphic transition from form IV to I occurred before the melting of form IV was complete. The temperature at which the solid converts from form IV to I is presumably reached by the instrument before the melting of form IV is complete. The heating rate clearly has a strong effect on the behavior of form IV. At 2 °C/min, there is sufficient time for the drug molecules to rearrange to form I between 113 and 120 °C, with no apparent melting. However, when the temperature reaches 120 °C (the melting onset of form IV), the polymorphic transition from form IV to I is not complete and the residual form IV in the sample begins to melt.

Melting is a thermodynamic event, which occurs much faster than recrystallization (a kinetic event). On the other hand, during a solid–solid polymorphic transition, the amount of each polymorphic form present changes at the same speed. The rate of change in the amount of each form of FFA present was analyzed as follows: a linear fit of the integrated data was carried out at the straightest sections of the phase fraction curves around the intersection (116.6–119.8 and 120–122 °C). It appears that the decline of form IV (−4.4946 °C^–1^) occurs at almost the same rate as the evolution of form I (4.0504 °C^–1^) (Figure S21a). The similarity between these two numbers indicates that the conversion is a continuous process and supports the conversion below 120 °C being a solid–solid transition. The same situation is observed from 120 to 122 °C, during which the rate of decrease of form IV (−2.1087 °C^–1^) is also similar to the growth of form I (2.1023 °C^–1^) (Figure S21b). This indicates that both before and after the start of form IV melting (120 °C), the form IV-to-form I transition proceeds via a solid-to-solid pathway, with no recrystallization from the molten FFA. The first endothermic peak in the DSC thermogram is thus the melting peak of the residual form IV (Figure S21c).

When pure amorphous FFA is heated at 2 °C/min, it only shows the crystallization and then the melting of form I (Figure S22). It is thus clear that making a composite with 4000 cp HPMC can drive FFA to recrystallize into the metastable form IV. This may be because drug molecules are dispersed in HPMC after cooling, and the viscosity of HPMC and H-bonds formed between FFA and HPMC provides resistance to the movement of drug molecules; thus, more time and energy are required for crystallization. Therefore, instead of recrystallizing directly into form I, the high-temperature stable form, the FFA molecules organize themselves to form the metastable form IV, which requires less energy and time. This has previously been noted with olanzapine and paracetamol ASDs in polymer carriers, for instance.^13,29^ With an increasing temperature (energy providing) and slow heating rate (sufficient time for transformation), molecules tend to rearrange to a more stable structure.

### FFA–100 000 cp HPMC Dispersions

DSC–XRD data on 5:1, 2:1, and 1:1 w/w formulations prepared with FFA–100 000 cp HPMC show that the samples recrystallize into form IV and then melt at 127 °C (Figures S23–S25 and Table S8). Data collected during reheating a 1:5 w/w FFA/HPMC 100 000 cp ASD illustrate that the sample remains almost completely amorphous during the heating process. A weak melting peak is observed at 126 °C, which likely corresponds to the melting of form IV,^28^ but there is too little crystalline material present to be detected by XRD (Figure S26).

### FFA–EC Dispersions

A 5:1 w/w FFA/EC ASD shows similar results to the 6 cp HPMC ASD at 5:1 w/w, with the sample first recrystallizing to form X and then converting to form IV (Figures S27–S29 and Table S9). FFA/EC dispersions made at 2:1 and 1:1 w/w polymers show similar phase transition behavior (Figures S30–S32 and Table S10). The first exothermic peak on the DSC trace appears at 60 °C, but no reflections are observed on the contour plot. This may be because during the first exothermic event, only a very small amount of sample crystallizes, below the detection limit for XRD. Bragg reflections of form IV begin to emerge at about 80 and 70 °C in the 2:1 and 1:1 w/w systems, respectively, coinciding with the onset of the second exothermic peak in the DSC traces. Following this, there is an endothermic event peaking at 126 °C in each thermogram, after which no Bragg reflections can be seen. Given the findings above, the latter peak is expected to be the melting of form IV.

For a dispersion of FFA/EC at a 1:5 w/w ratio (Figure S33), there are almost no reflections shown in the contour plot and no endothermic events exist in the DSC trace, suggesting that the sample remains amorphous during heating. There is a distinct reflection present at 0.8° from 0 to 160 °C; the origin of this is not clear, but it is thought to arise from some impurities in the sample.

### FTIR

**Figure 6:**
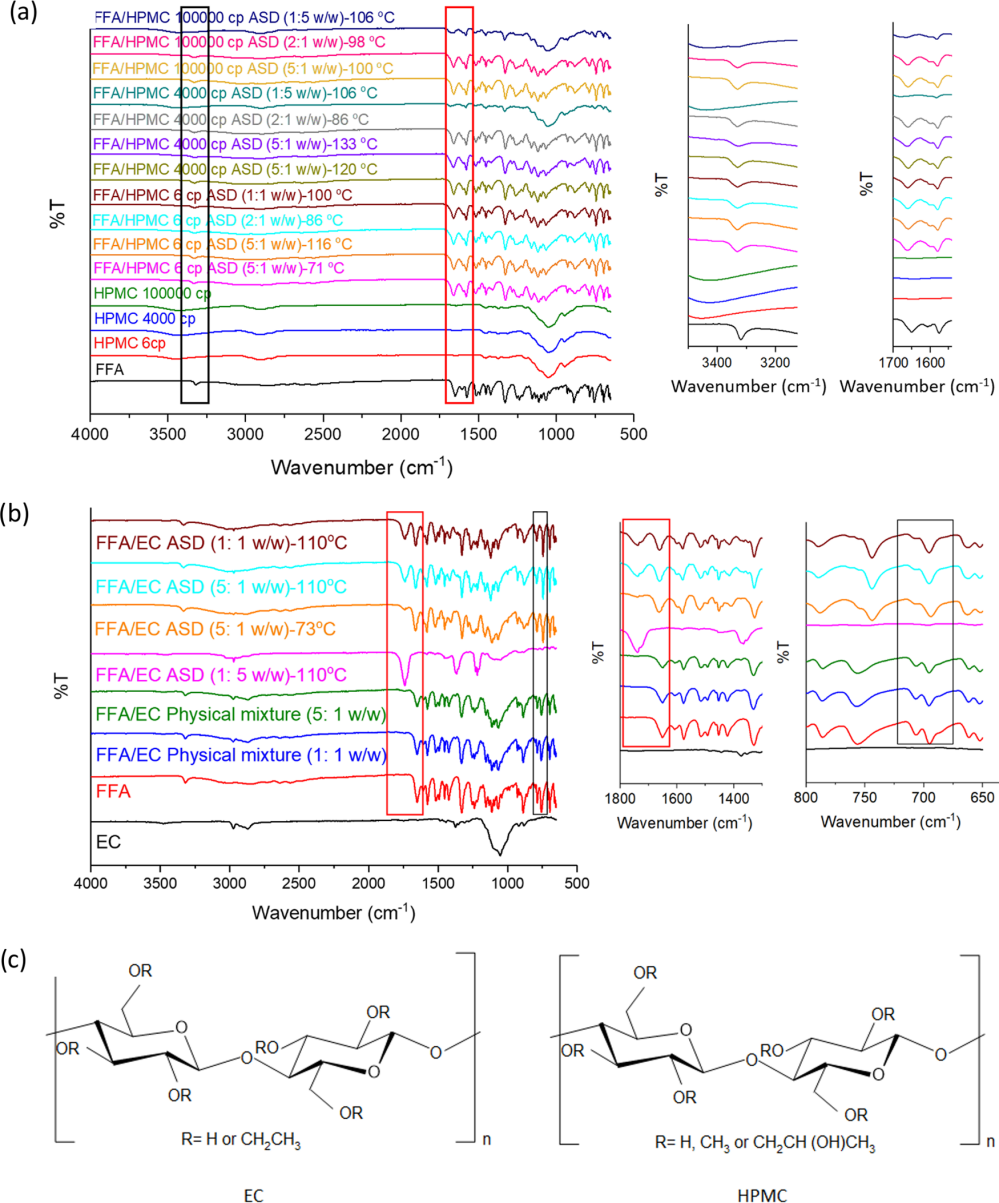
FTIR spectra for (a) FFA, HPMC, and their ASDs after recrystallization; (b) FTIR spectra for FFA, EC, a physical mixture, and FFA/EC ASDs at 73 and 110 °C; and (c) chemical structures of EC and HPMC.

The drug/polymer interactions were studied by FTIR. All of the samples were heated up in the DSC and then removed for FTIR characterization. The FTIR spectra are presented in Figure 6. FFA showed a characteristic peak due to the stretching of the secondary amine N–H at 3318 cm^–1^. The C=O and C=C vibrations are at 1654 and 1578 cm^–1^, respectively.^30^ For FFA/HPMC after heating, a minor shift in the N–H group at 3333 cm^–1^ was recorded, which might indicate the formation of hydrogen bonding between the amine group in FFA and the hydroxyl groups in HPMC. No shifts in the vibration bands of the carbonyl functional groups were found in the FFA/HPMC systems.

For FFA/EC, the most obvious difference between the spectra of the formulations and raw materials is the peak at ca. 1740 cm^–1^, which is only present in the ASD systems. The peak at 1660 cm^–1^ present before heating is the C=O vibration of FFA. After heating, an H-bonding interaction presumably arose between the carboxyl of FFA and the −OCH_2_CH_3_ group of EC, which causes a large shift in the C=O vibration to 1740 cm^–1^. For the FFA/EC 1:5 w/w ASD, the only C=O peak is at 1740 cm^–1^, and no crystallization occurred at this drug/polymer ratio. This suggests that the formation of H-bonds inhibits the crystallization of FFA, making it stay in the amorphous form. Peaks at both 1660 and 1740 cm^–1^ exist in the spectra of the 5:1 and 1:1 w/w samples, which suggests that some carboxyl groups of FFA are not forming H-bonds with EC at these drug–polymer ratios. Therefore, FFA molecules in these systems are still able to pack into crystals. The C–F vibrations of the ASDs at 744 and 694 cm^–1^ show red shifts compared with the FFA pure material, suggesting that H-bonds are forming here too in the ASD samples. All of the changes described are consistent with a change in hydrogen-bonding interactions between FFA and EC during heating, which affects the polymorphic transition of these ASDs. The structures of EC and HPMC are shown in Figure 6c. The structures indicate that HPMC is more hydrophilic than EC (has more −OH group); in the dispersions, these hydroxyl groups on HPMC interact with the amine groups of FFA. This interaction presumably affects the steric position between the methoxy (HPMC) and carboxyl groups (FFA), inhibiting the interaction between these two groups. Therefore, no shift of the C=O peak is observed in Figure 6a. In contrast, no peak shift of the amine group was observed in the FFA/EC systems: instead, the H-bond arose between the carboxyl of FFA and the ethoxy group of EC.

## Discussion

The three different grades of HPMC obtained vary in viscosity, with values of 6, 4000, and 100 000 cp, respectively. HPMC is a methyl and hydroxypropyl mixed cellulose; of the grades of HPMC used in this study, the two HMPC 2208 samples have the same substitution type, with 19–24% methoxy groups and 4–12% hydroxypropyl groups, while the HPMC 2910 material contains 28–30% methoxy groups and 7–12% hydroxypropyl groups (Table 2).^31^

**Table 2:** Summary of the Key Properties of the Different HPMC Grades

sample name	viscosity (cp)	methoxy (%)	hydroxypropyl (%)
HPMC 2910 (PharmaCoat 606)	6	28–30	7–12
HPMC 2208 (K4M)	4000	19–24	4–12
HPMC 2208 (K100M)	100 000		

The XRD–DSC results for FFA/HPMC ASDs show that different grades and ratios of HPMC cause different phase transition processes to occur. For HPMC with a viscosity of 4000 and 100 000 cp, when the polymer ratio is low (FFA/HPMC > 1:5 w/w), the polymer content of the blend does not lead to a huge difference and the samples show the same phase transition properties. Compared with pure FFA, which experienced three different transitions and finally recrystallizes into form I under the same heating conditions, FFA with HPMC 100 000 cp recrystallizes to form IV, while FFA with HPMC 4000 cp recrystallizes to form IV and then converts to form I. The polymorphic transitions for FFA/HPMC 6 cp ASD are somewhat different; when the drug/polymer ratio is 5:1 w/w, it recrystallizes into an unknown phase (“pattern X”) before converting to form IV. The transition with the 2:1 w/w 6 cp HPMC ASD is the same as that of 100 000 cp ASD, while FFA remains almost completely amorphous when the drug/polymer ratio is 1:1 w/w. Additionally, for any HPMC grade at a 1:5 w/w drug/polymer ratio, the large amount of polymer present provides significant steric hindrance to molecular motion, and the FFA remains largely amorphous throughout the heating cycle.

Considering the effects of different types of HPMC, at low HPMC contents (drug/polymer > 1:5 w/w), the system with HPMC 4000 cp first recrystallizes to form IV, which then converts into form I through a mechanism involving localized melting. However, the analogous material made with HPMC 100 000 cp only recrystallized into form IV. This result suggests that the higher viscosity of HPMC can stabilize metastable form IV during heating, presumably because it leads to greater steric hindrance to molecular mobility. The HPMC 6 cp composite (2:1 w/w) displays the same phase transition properties as that made with HPMC 100 000 cp, however (and differs from the 4000 cp analogues). The viscosity of HPMC 6 cp is ca. 15 000 times lower than that of HPMC 100 000 cp, but there is also a difference in the amount of methoxy and hydroxypropyl groups present, with HPMC 6 cp having rather more of these. Both types of HPMC show the same inhibition effects on the polymorphic transitions of FFA, which might suggest that both viscosity and the substitution have an effect on the transformation. HPMC 6 cp has more methoxy and hydroxypropyl groups, making it more branched; thus, it gives similar steric hindrance to HPMC 100 000 cp, even though the latter is much higher in viscosity.

**Figure 7:**
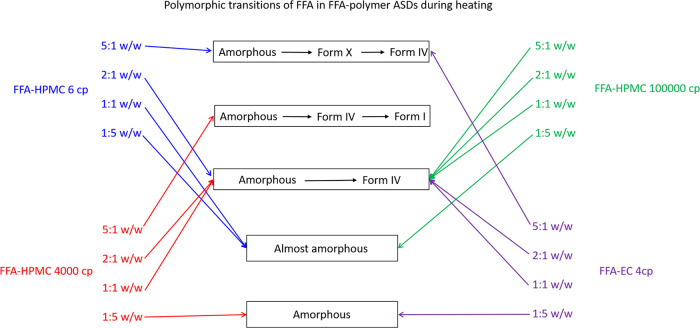
Diagram summarizing the crystallization pathway of FFA in FFA/HPLC and EC ASDs, showing the major polymorphic transitions observed in systems prepared with different polymer grades and varied drug loadings.

The polymorphic transitions of the FFA/EC 4 cp ASDs are similar to those with FFA/HPMC 6 cp ASDs, except for the 1:1 w/w FFA: polymer ratio where with HPMC the FFA remains amorphous during heating, while the EC ASD recrystallizes into form IV. A summary of the polymorphic transition with the different ASDs is shown in Figure 7. Together with the HPMC data, these findings suggest that the chemical substitution of a polymer plays a significant role in polymorphic transitions and is arguably more influential than the viscosity of the polymer. EC is a hydrophobic polymer, while HPMC is hydrophilic. It is expected that EC has a higher affinity with FFA since both of them are hydrophobic, which may result in a stronger inhibition of FFA molecular movement during heating; FTIR confirms that more H-bonding occurs with EC than with HPMC. For ASDs at 1:5 w/w, the FFA/HPMC systems remain almost amorphous during heating, but still a small endothermic peak can be observed. The FFA/EC system is completely amorphous since no reflections or melting peak can be seen in XRD–DSC. This result confirms that the hydrophobic EC polymer inhibits more strongly the molecular movement of FFA during heating. The FTIR studies of FFA/EC ASDs also suggest that the formation of H-bonds between FFA and the polymer has a significant effect on the polymorphic transitions, which again confirms the importance of the chemical substitution of polymer.

## Conclusions

The aim of this work was to study heat-induced crystallization and polymorphic phase transitions in FFA–polymer ASDs. We have shown that FFA forms X, IV, I, and amorphous material are all obtained upon heating the ASDs. This study shows that FFA form IV can be stabilized by HPMC 6 and 100 000 cp and EC 4 cp, which inhibit the transition from form IV to I during heating. Increasing the polymer content of the ASD also inhibits polymorphic transitions, with drug/polymer ratios of 1:5 w/w, resulting in FFA remaining largely amorphous during heating.

Comparing FFA–HPMC 6 and 100 000 cp dispersions suggested that the substitution type has a more distinct impact on the polymorphic stability than the viscosity of HPMC. These two HPMCs have different substitution types, with the 6 cp material having notably more methoxy and hydroxypropyl side groups and thus being more branched. ASDs with these two types of HPMCs experience largely the same phase transitions. In contrast, ASDs made with the 4000 cp HPMC (which has the same substitution pattern as 100 000 cp) show a reduced stabilization of metastable polymorphs. These findings suggest that the increased presence of methoxy and hydroxypropyl groups aids HPMC with low viscosity to have the same stabilizing effects as much more viscous systems. Comparing the HPMC data with findings in the FFA/EC system, it is again clear that the chemical substitution of the polymer plays a more significant role in directing polymorphic transitions than the viscosity.

## Supporting Information Available

Additional crystallographic and XRD–DSC data for FFA and FFA–polymer dispersions (PDF)
