# Supplementary material for: Monitoring Polymorphic Phase Transitions in Flufenamic Acid Amorphous Solid Dispersions Using Hyphenated X-ray Diffraction–Differential Scanning Calorimetry
Source: Mol Pharm. 2022 Mar 29;19(5):1477–87. doi: 10.1021/acs.molpharmaceut.2c00016 (PMC9097533; doi:10.1021/acs.molpharmaceut.2c00016)
Supplement: Supplementary file 1 — mp2c00016_si_001.pdf [file mp2c00016_si_001.pdf]

S1. CRYSTALLOGRAPHIC DATA FOR FFA

Table S1. Crystallographic data for FFA form I to form VIII.

Form	I	II	III	IV	V	VI	VII	VIII
**CSD ID**	FPAMCA 11	FPAMCA 17	FPAMCA	FPAMC A15	FPAMCA 16	FPAMC A14	FPAMC A12	FPAMC A13
**Crystal system**	monoclinic	monoclinic	monoclinic	triclinic	monoclinic	triclinic	triclinic	triclinic
**Space group**	$P2_1/c$	$P2_1/c$	C2/c	P-1	$P2_1/c$	P1	$P2_1/c$	P-1
**Temperature (K)**	300	95	300	273	95	85	85	85
**a/ Å**	12.523(4)	10.8813 (8)	39.84	8.7589 (2)	26.6592 (18)	8.6485 (2)	14.967 (14)	17.007 (14)
**b/ Å**	7.868(6)	10.2374 (7)	5.10	11.6629 (3)	7.9007(2)	11.5115 (2)	20.641 (2)	17.880 (14)
**c/ Å**	12.874(3)	11.7487 (11)	12.24	20.0229 (14)	23.2430(5)	38.895 (3)	7.9486 (8)	19.191 (15)
**α/ °**	90	90	90	80.632 (6)	90	87.914 (6)	90	81.321(7)
**β/ °**	95.2(2)	111.318 (8)	92.47	81.041 (6)	94.084(7)	85.910 (6)	98.316 (7)	89.580(6)
**γ/ °**	90	90	90	73.534 (5)	90	72.260 (5)	90	78.560(7)
**Cell volume (Å)**	1263.27	1219.21	2488.57	1922.33	4883.20	3678.32	2430.1	5655.09
**Density (g/cm³)**	1.470	1.532	-	1.458	1.530	1.523	1.537	1.569
**Z**	4	4	8	6	16	12	8	19
**Z'**	1	1	1	3	4	6	2	9.5

S2. PURE FFA

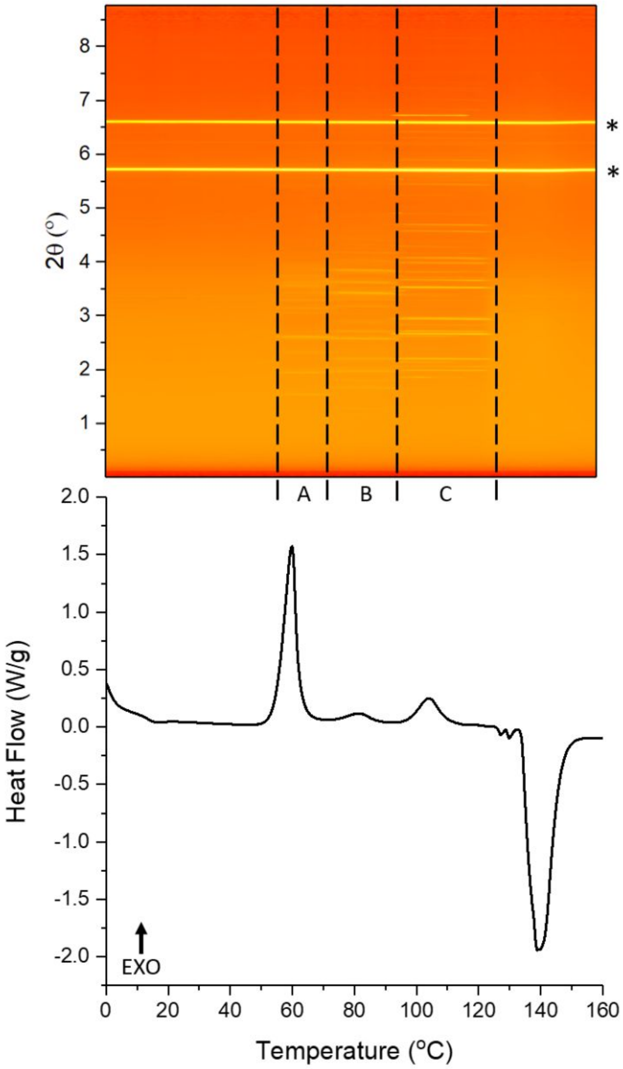

Figure S1. DSC-XRD data for pure FFA on the second heat, after heating FFA form I to melting and then cooling to 0 °C (reflections marked * are a result of the aluminium pan). The lack of initial Bragg reflections illustrates that the starting material is amorphous, as expected after quench cooling. The first exothermic peak in the DSC trace occurs at 54 °C (generating "form X"), with reflections appearing in XRD at the same temperature and showing this to arise from recrystallisation. There are then two exothermic peaks at 74 °C (conversion of form X to form IV) and 97 °C (transformation of form IV to form I), which are coincident with changes in the positions of the Bragg reflections in the XRD data. Two very small endotherms are observed at 127 °C and 129 °C; following these, at 133 °C, there is a much larger endothermic event coinciding with the total loss of Bragg reflections in the contour plot and thus attributed to melting.

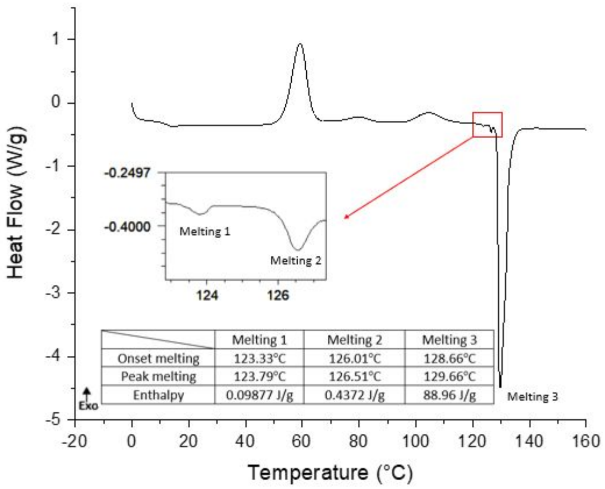

Figure S2. DSC profile of amorphous FFA heated at 10°C/min.

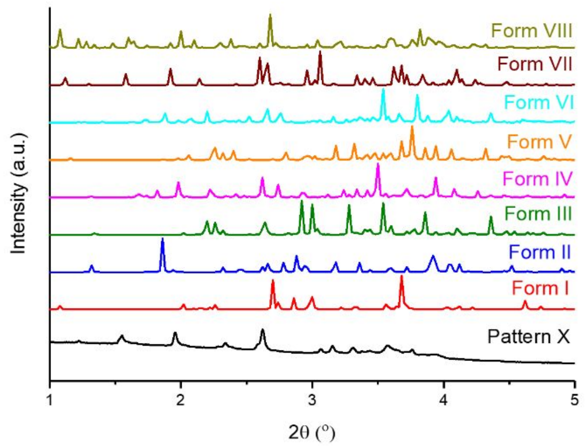

Figure S3. Comparison of pattern X to those calculated for FFA form I to form VIII using data from the CSD (wavelength 0.234 Å).

Table S2: Characteristic Bragg reflections (λ = 0.234 Å) for FFA form X.

2θ (°)	Intensity (a.u.)
1.22	63.35
1.44	50.54
1.54	152.87
1.65	67.21
1.80	57.59
1.94	244.17
2.20	70.13
2.26	76.73
2.32	132.33
2.36	75.01
2.43	63.51
2.48	60.70
2.60	340.94
2.70	79.23
2.89	58.66
3.04	121.06
3.12	166.62
3.28	152.29
3.41	160.23
3.54	193.24
3.61	166.69
3.68	131.04
3.72	157.29
3.84	129.20
4.01	75.09
4.16	69.09
4.40	59.07
4.58	52.83
4.67	51.77
4.83	49.94
4.96	49.88

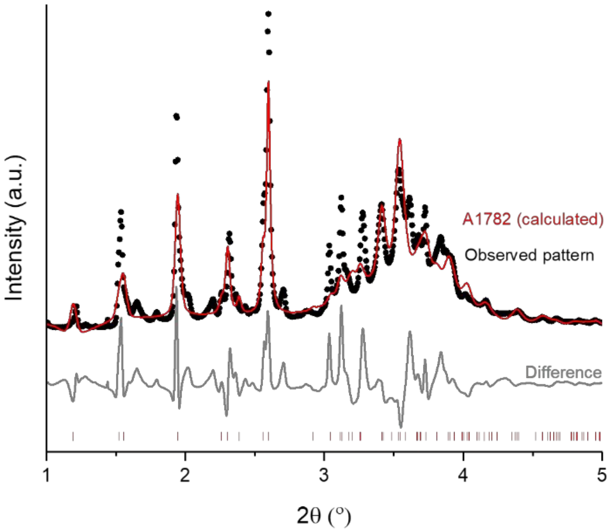

Figure S4. Rietveld refinement of predicted FFA structure A1782[1] against a diffraction pattern of form X, recorded after heating an FFA glass from 0 to 68°C at 10 °C/min. Tick marks show the positions of allowed reflections from FFA A1782.

Table S3. Refinement parameters for computationally predicted FFA A1782 against observed data for form X.

**Form**	**A1782**
**Temperature (°C)**	68
**Space group**	A2/n
**a/ Å**	22.474(12)
**b/ Å**	4.2362(23)
**c/ Å**	27.571(15)
**α/ °**	90
**β/ °**	88.715(57)
**γ/ °**	90
**R_{wp}**	18.116
**Phase fraction**	100%

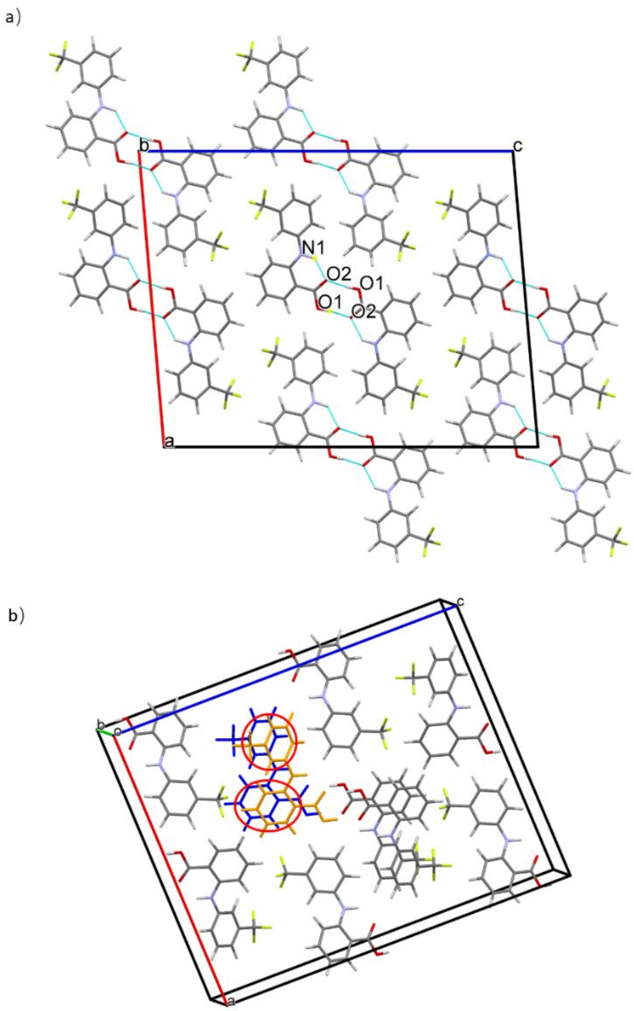

Figure S5. a) Computationally predicted FFA A1782 viewed in the ac plane of the unit cell. b) π··· π interactions in FFA A1782 viewed down the b direction (the benzene rings with π··· π interactions are marked by red circles). In this structure, hydrogen bonds between the hydrogen on O1 and O2 of an adjacent molecule (O2···H—O1; 1.696 Å) hold the FFA molecules together in dimers, and stabilise the unit cell in the ac plane. In the b direction, adjacent dimers are stabilised through week π··· π interactions.

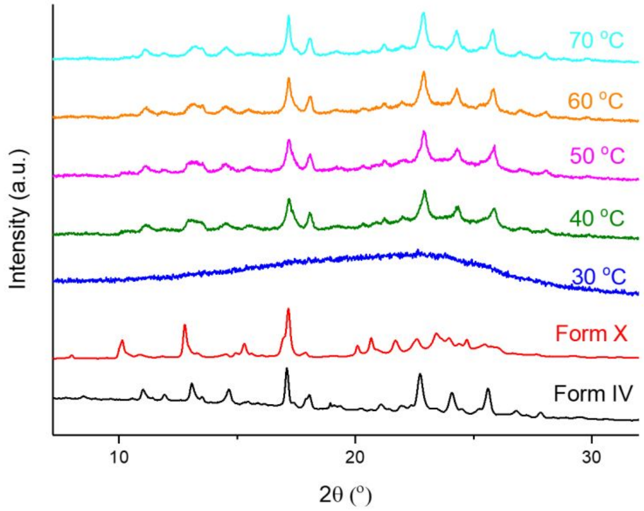

Figure S6. The XRD patterns of FFA form IV, form X and patterns recorded by VT-XRD (FFA glass prepared by melting and cooling with ice water, wavelength: 1.5406 Å).

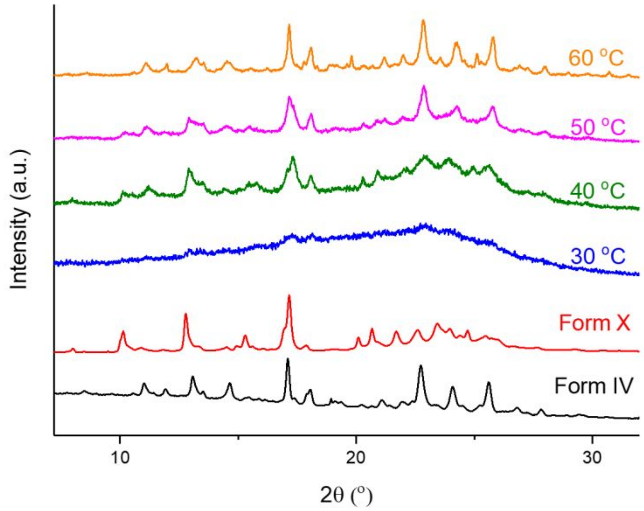

Figure S7. The XRD patterns of FFA form IV, form X and patterns recorded by VT-XRD (FFA glass prepared by melting and cooling with an Oxford Instruments CryojetHT, wavelength: 1.5406 Å).

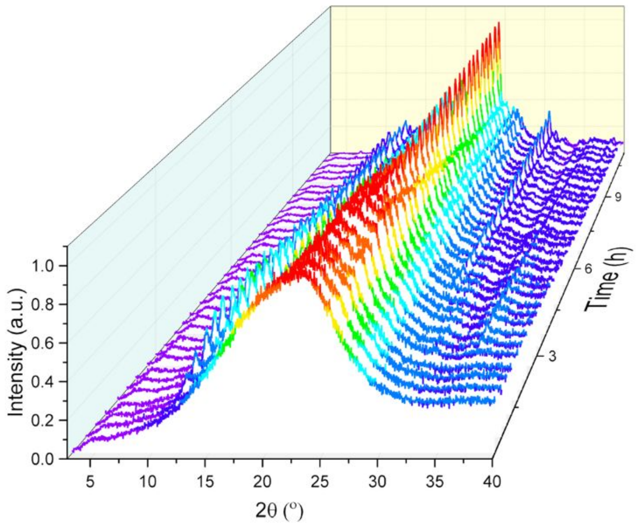

Figure S8. The XRD patterns obtained on an FFA glass allowed to cool from 145 ºC to room temperature in an XRD over 11 h.

S3. 5:1 w/w FFA/ HPMC (6 cp) DISPERSION

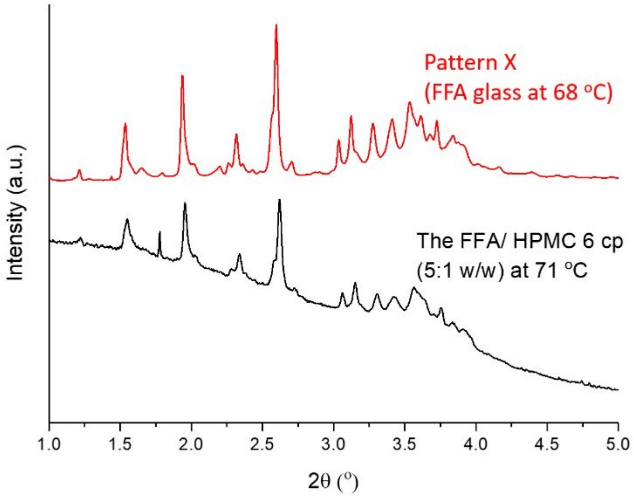

Figure S9. Overlay of pattern X obtained for FFA alone and the observed XRD pattern of the 5:1 w/w FFA/HPMC 6 cp ASD at 71 °C (on reheating).

S4. 2:1 w/w FFA/ HPMC (6 cp) DISPERSION

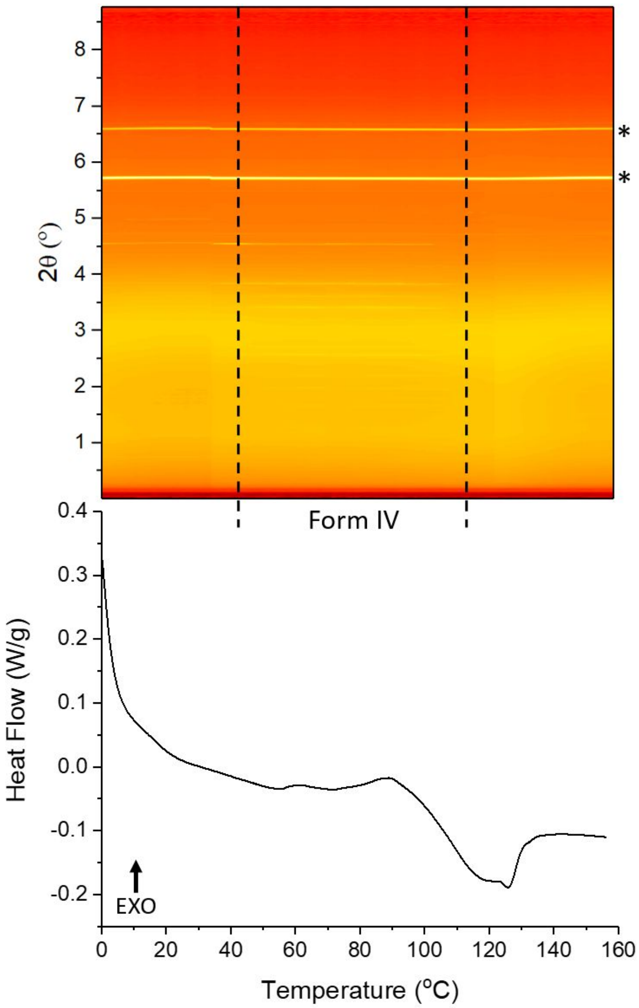

Figure S10. XRD-DSC data obtained during the reheating of the 2:1 w/w FFA/HPMC 6 cp mixture. Reflections marked * are a result of the aluminium pan.

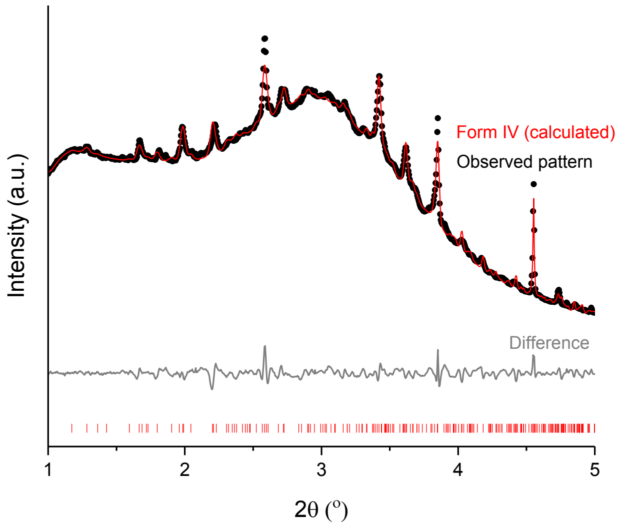

Figure S11. Rietveld refinement against the diffraction pattern recorded at 86 °C during the reheating of the 2:1 w/w FFA/HPMC 6 cp mixture; tick marks show the position of allowed reflections of FFA form IV(FPAMCA15).

Table S4.Refinement parameters for the FFA/HPMC 6 cp ASD (2:1 w/w) at 86 °C. The starting model was taken from the CSD (form IV: FPAMCA15).

**Form**	**IV**
**Temperature (°C)**	86
**Space group**	P-1
**a/ Å**	8.7948(15)
**b/ Å**	11.968(1)
**c/ Å**	20.024(6)
**α/ °**	81.010(16)
**β/ °**	81.828(18)
**γ/ °**	74.107(15)
**R_{wp}**	1.6701
**Phase fraction[a]**	76%

[a]The representative error of the phase fractions cannot be calculated because of the graininess of the sample.

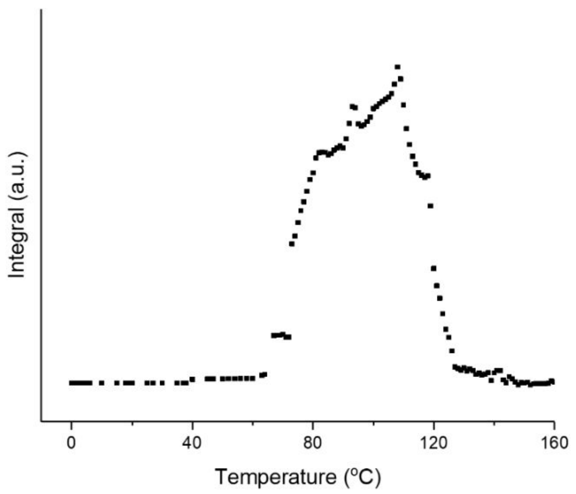

Figure S12. Plot of integrated total diffracted intensity from FFA form IV as a function of temperature in the 2:1 w/w FFA/HPMC 6 cp mixture.

S5. 1:1 AND 1:5 w/w FFA/ HPMC (6 cp) DISPERSIONS

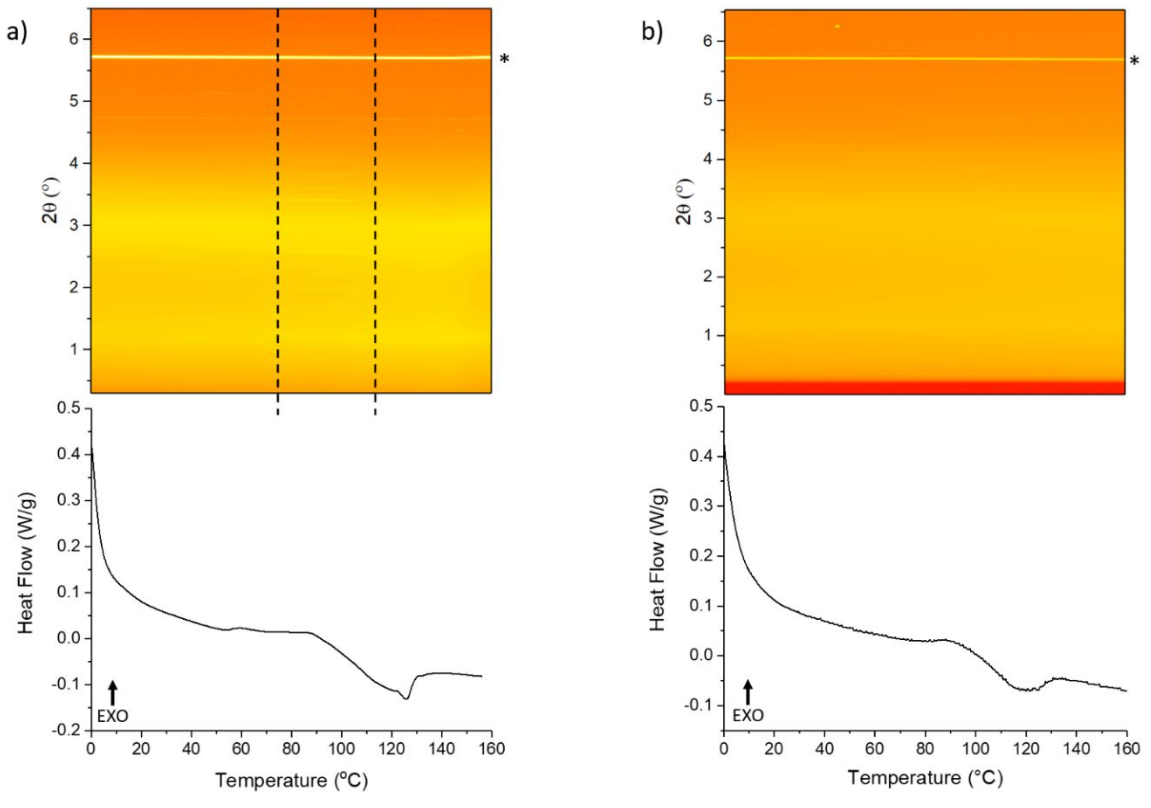

Figure S13. XRD-DSC data collected during the reheating of FFA/HPMC 6 cp ASDs: a) 1:1 w/w; b) 1:5 w/w. Reflections marked * are from the aluminium pan.

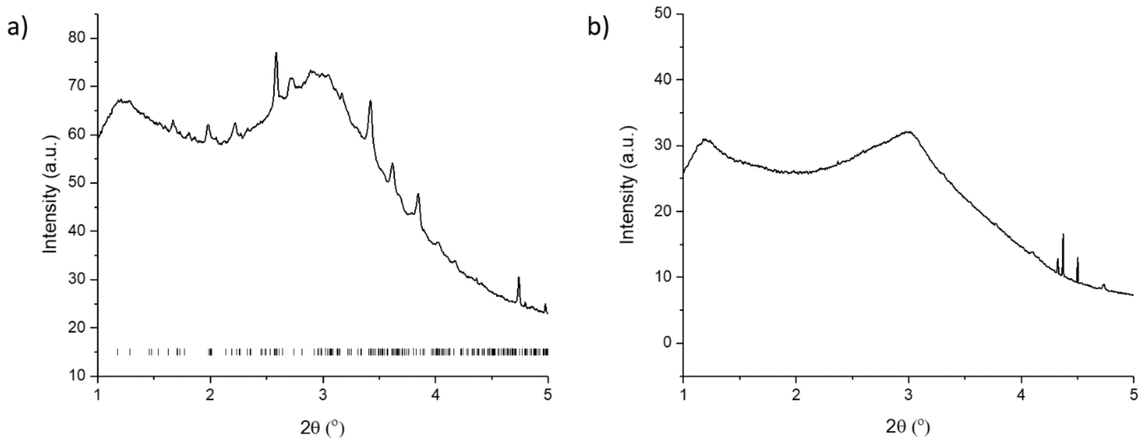

Figure S14. XRD patterns obtained at 100 °C during the reheating of FFA/HPMC 6 cp ASDs. a) 1:1 w/w, tick marks show the position of allowed reflections of FFA form IV (FPAMCA15); b) 1:5 w/w.

S7. 5:1 w/w FFA/ HPMC (4000 cp) DISPERSION

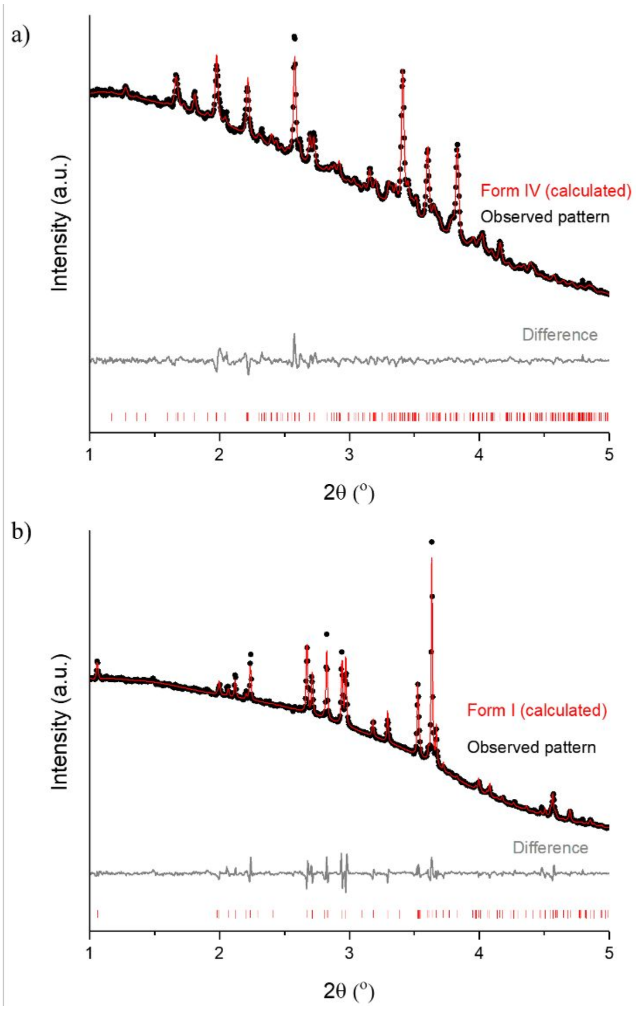

Figure S15. Rietveld refinement against diffraction patterns collected on a 5:1 w/w FFA/ HPMC 4000 cp ASD at a) 120 °C; tick marks show the position of allowed reflections of FFA form IV (FPAMCA15); b) 133 °C; tick marks show the position of allowed reflections of FFA form I (FPAMCA11).

Table S5. Refinement parameters obtained during heating a FFA/HPMC 4000 cp ASD (5:1 w/w). The starting models were taken from the CSD (form I: FPAMCA11, form IV: FPAMCA15).

**Form**	**IV**	**I**
**Temperature (°C)**	120	133
**Space group**	P-1	P21/c
**a/ Å**	8.7825(14)	12.698(1)
**b/ Å**	12.005(1)	7.9211(1)
**c/ Å**	20.092(3)	13.021(2)
**α/ °**	80.491(12)	90
**β/ °**	81.074(12)	95.176(5)
**γ/ °**	74.072(7)	90
**R_{wp}**	1.1494	1.4095
**Phase fraction[a]**	100%	90%

[a]The representative error of the phase fractions cannot be calculated because of the graininess of the sample.

S6. 2:1 AND 1:1 w/w FFA/ HPMC (4000 cp) DISPERSIONS

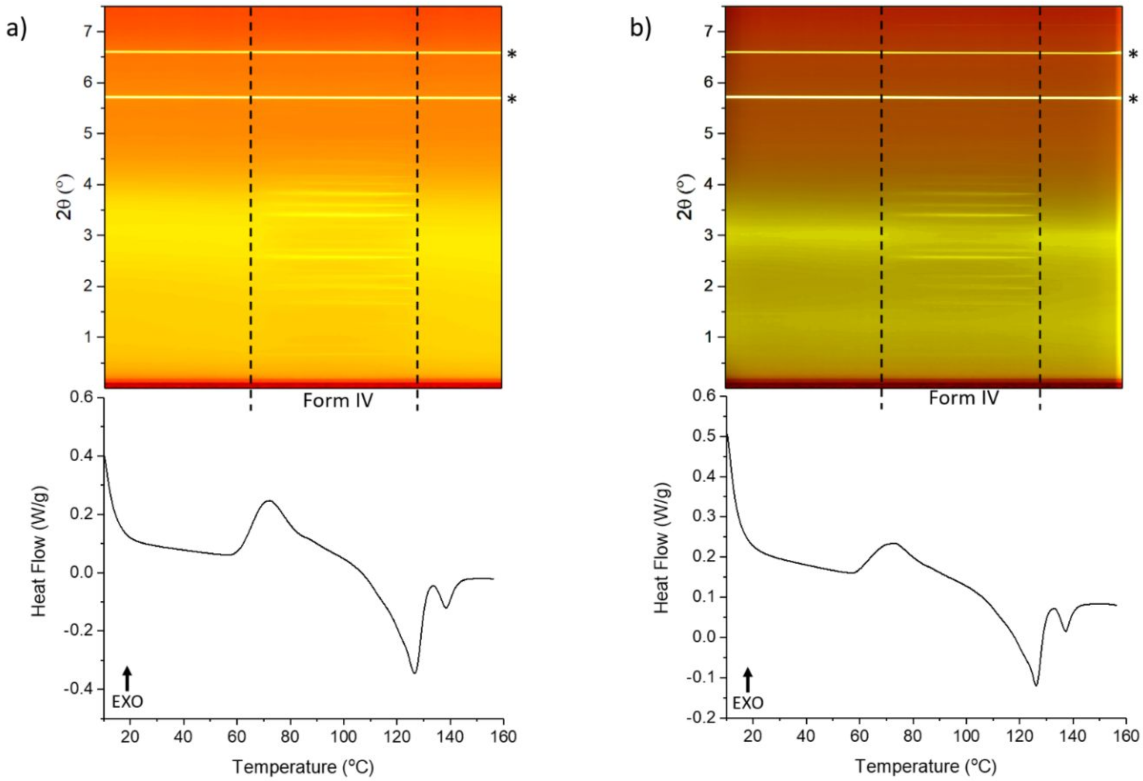

Figure S16. XRD-DSC data for the reheating of FFA/HPMC 4000 cp ASDs prepared at a) 2:1 w/w and b) 1:1 w/w. Reflections marked * are a result of the aluminium pan.

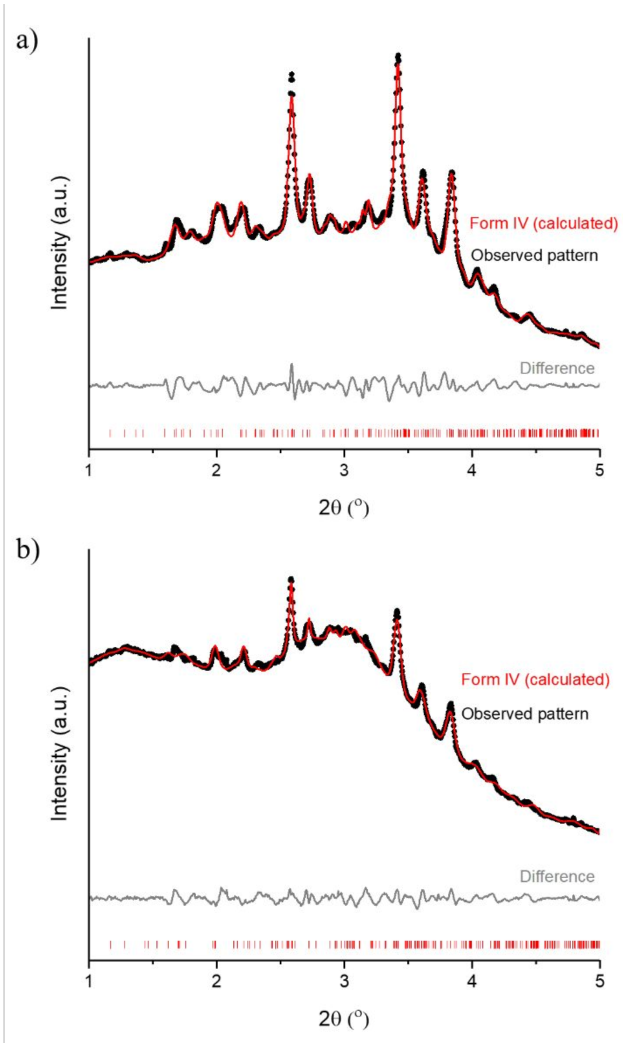

Figure S17. Rietveld refinement for diffraction patters recorded during heating of FFA/HPMC 4000 cp ASDs. a) 2:1 w/w at 86 ^{o}C; b) 1:1 w/w at 100 ^{o}C. Tick marks show the position of allowed reflections of FFA form IV (FPAMCA15).

Table S6. Refinement parameters for patterns obtained on heating FFA/HPMC 4000 cp ASDs (2:1 and 1:1 w/w) at 100 ^{o}C. The starting model was taken from the CSD (form IV: FPAMCA15).

**Form**	**IV (2:1 w/w)**	**IV (1:1 w/w)**
**Temperature (^{o}C)**	86	100
**Space group**	P-1	P-1
**a/ Å**	8.7720(38)	8.8725(15)
**b/ Å**	11.993(2)	11.991(3)
**c/ Å**	19.964(6)	19.026(15)
**α/ ^{o}**	81.360(27)	79.392(44)
**β/ ^{o}**	82.600(34)	81.520(48)
**γ/ ^{o}**	74.516(20)	75.300(35)
**R_{wp}**	2.3516	1.3664
**Phase fraction[a]**	71%	100%

[a]The representative error of the phase fractions cannot be calculated because of the graininess of the sample.

S7. 1:5 w/w FFA/ HPMC (4000 cp) DISPERSION

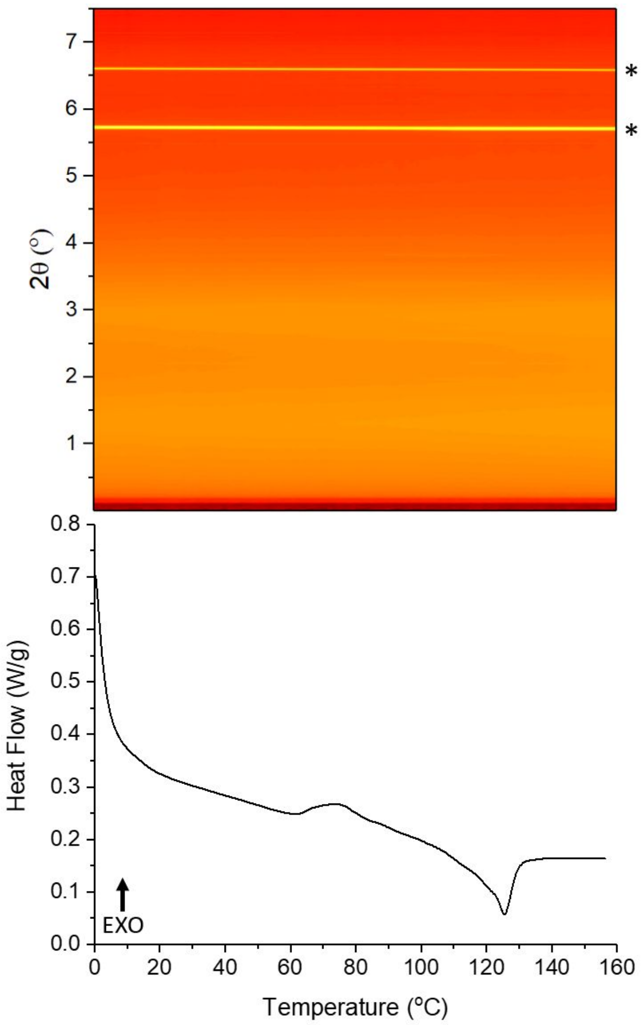

Figure S18. XRD-DSC data for the reheating of a FFA/HPMC 4000 cp ASD prepared at 1:5 w/w. Reflections marked * are a result of aluminium pan.

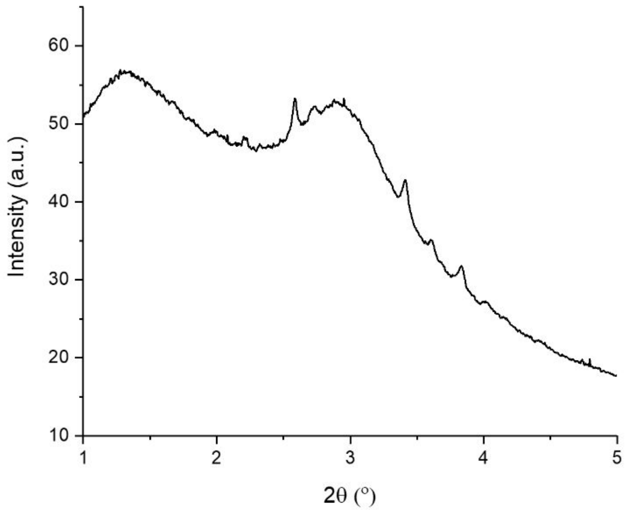

Figure S19. XRD pattern obtained at 106°C while heating the 1:5 w/w FFA/HPMC 4000 cp ASD.

S8. 1:1 w/w FFA/ HPMC (4000 cp) DISPERSION HEATED AT 2 ^{o}C/min

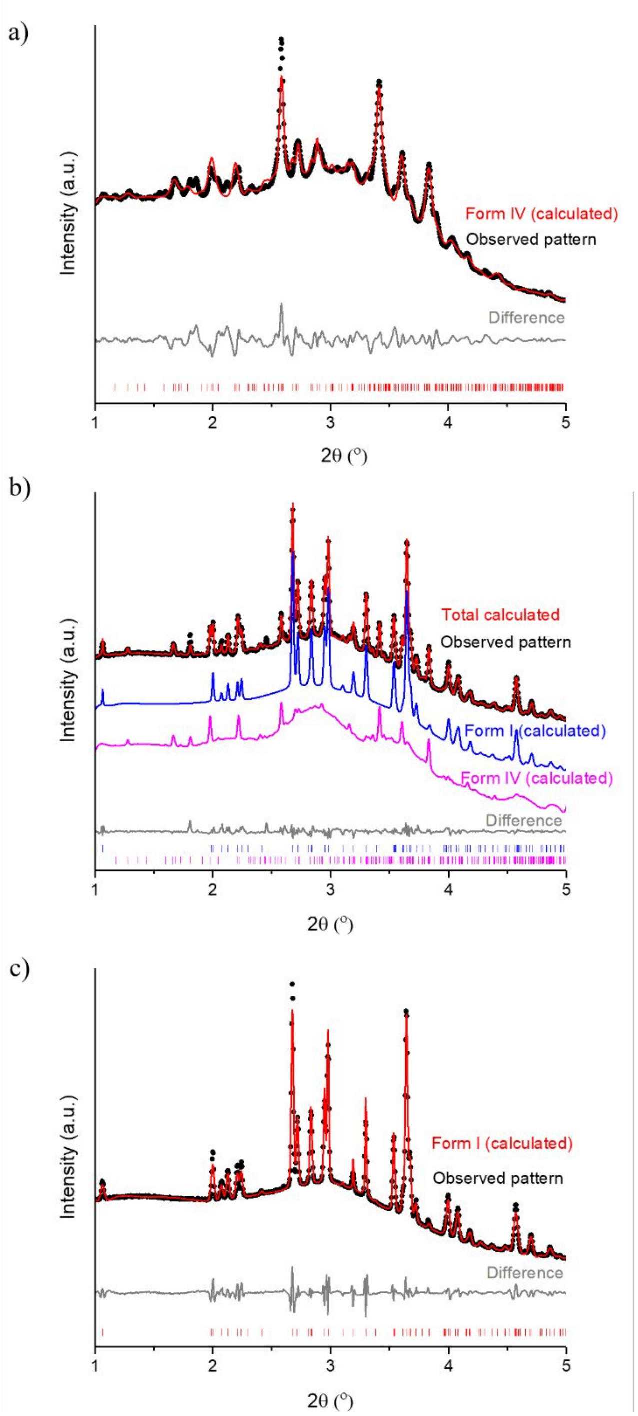

Figure S20. Rietveld refinement for a 1:1 w/w FFA/HPMC 4000 cp ASD during heating at 2 ^{o}C/min, showing patterns obtained a) at 95 ^{o}C, tick marks show the position of allowed reflections of FFA form IV; b) at 117 ^{o}C, tick marks show the position of allowed reflections of FFA form I (upper), form IV (lower); c) at 125 ^{o}C, tick marks show the position of allowed reflections of FFA form I.

Table S7. Refinement parameters for FFA in a 1:1 w/w FFA/HPMC 4000 cp ASD heated at 2 °C/min. The starting models were taken from the CSD (form I: FPAMCA11, form IV: FPAMCA15).

**Form**	**IV**	**I**	**IV**	**I**
**Temperature (°C)**	100	117		125
**Space group**	P-1	P21/c	P-1	P21/c
**a/ Å**	8.8027(56)	12.646(1)	8.7810(27)	12.657(1)
**b/ Å**	12.002(2)	7.9130(7)	11.985(1)	7.9247(9)
**c/ Å**	19.989(10)	12.987(1)	20.074(6)	12.994(1)
**α/ °**	81.234(35)	90	80.370(24)	90
**β/ °**	82.369(38)	95.198(5)	81.017(20)	95.231(5)
**γ/ °**	74.316(29)	90	74.078(15)	90
**R_{wp}**	2.8282	1.5631		2.9921
**Phase fraction[a]**	97%	65%	35%	97%

[a]The representative error of the phase fractions cannot be calculated because of the graininess of the sample.

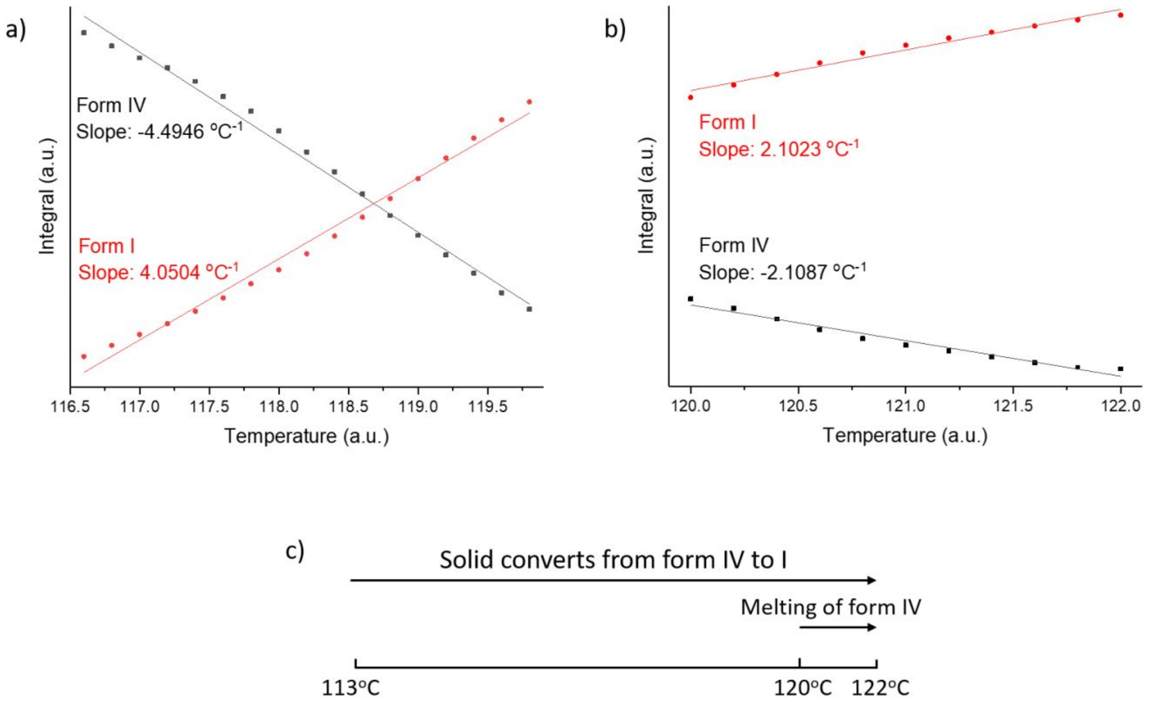

Figure S21. Plot of integrated total diffracted intensity for FFA form I and form IV in a 1:1 w/w FFA/ HPMC 4000 cp ASD heated at 2 ^{o}C/min over a) 116.2 to 118.6 ^{o}C; b) 120 to 122 ^{o}C ; c) diagram illustrating the transition process from 113 to 122 ^{o}C.

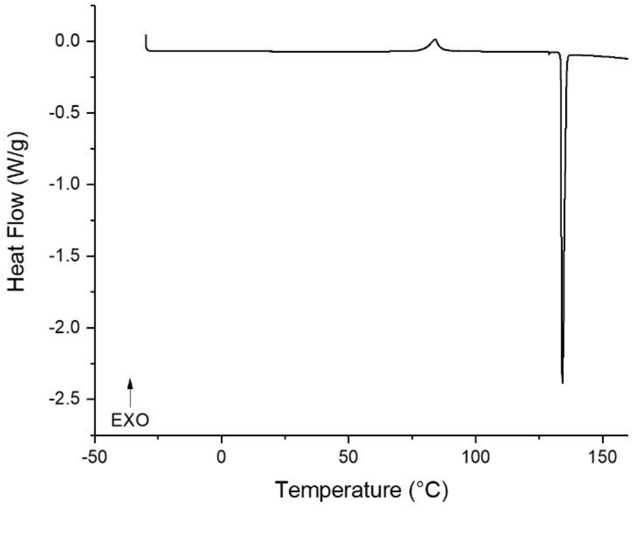

Figure S22. DSC profile of amorphous FFA reheated at 2 ^{o}C/min.

S9. 5:1, 2:1 AND 1:1 w/w FFA/ HPMC (100000 cp) DISPERSIONS

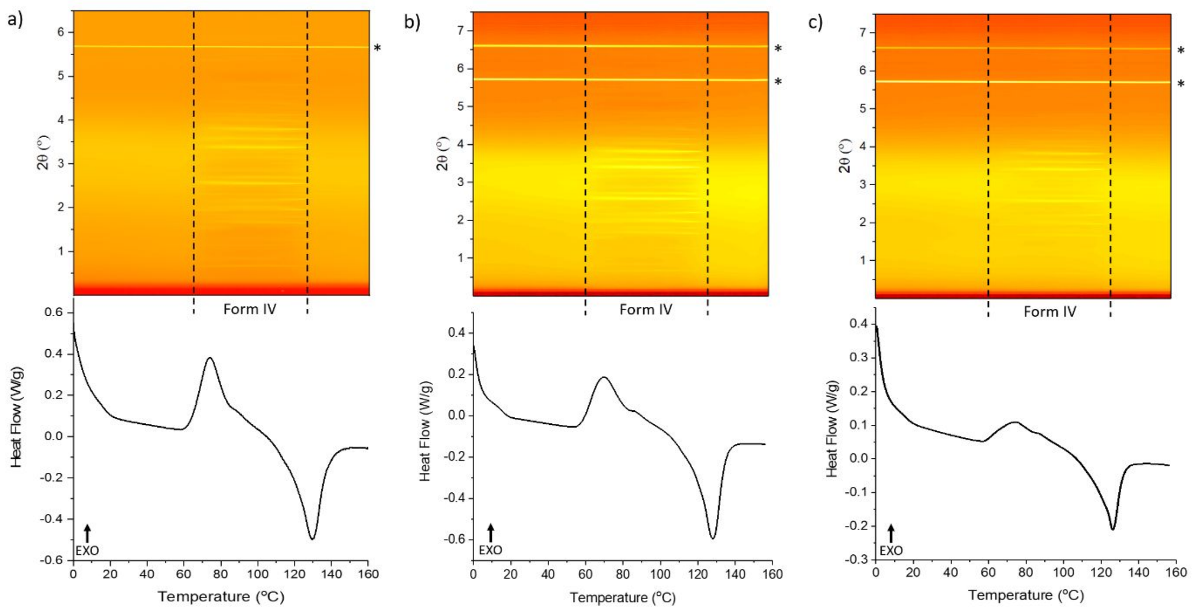

Figure S23. XRD-DSC data for the reheating of FFA/ HPMC 100000 cp ASDs prepared at a) 5:1 w/w, b) 2:1 w/w, c) 1:1 w/w. Reflections marked * are a result of the aluminium pan. Bragg reflections of form IV first appear at 65 ^{o}C (5:1 w/w) or 60 ^{o}C (2:1 and 1:1 w/w), corresponding to the onset temperature of the exothermic peak in the DSC trace. Following this, there is an endothermic event peaking at 127 ^{o}C (5:1 w/w) or 126 ^{o}C (2:1 and 1:1 w/w), at which a total loss of Bragg reflections is observed. This second endotherm therefore corresponds to the melting of FFA, and is in agreement with the literature values for form IV.[2]

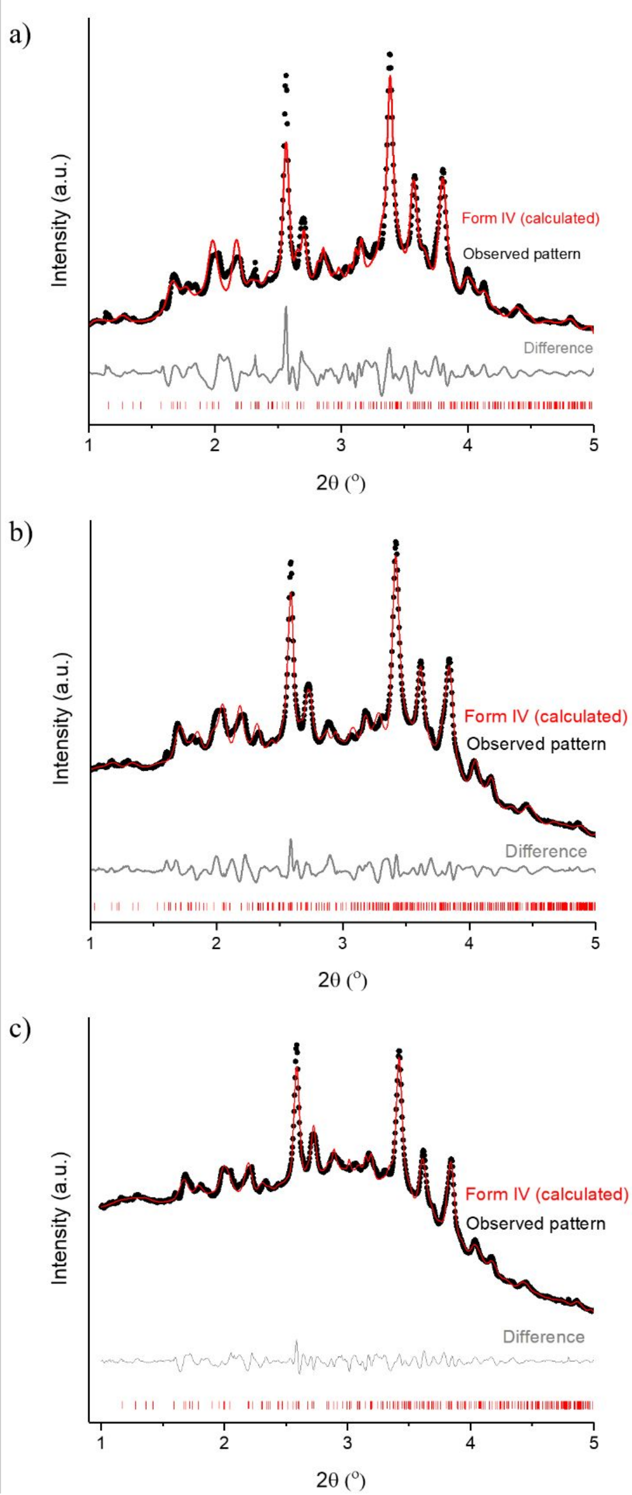

Figure S24. Rietveld refinements on diffraction patterns recorded during reheating FFA/ HPMC 100000 cp ASDs. a) 5:1 w/w at 100 °C; b) 2:1 w/w at 98 °C; c) 1:1 w/w at 101 °C. Tick marks show the position of allowed reflections of FFA form IV (FPAMCA15).

Table S8. Refinement parameters calculated during reheating FFA / HPMC 100000 cp ASDs. The starting model was taken from the CSD (form IV: FPAMCA15).

**Form**	**IV (5:1 w/w)**	**IV (2:1 w/w)**	**IV (1:1 w/w)**
**Temperature (°C)**	100	98	101
**Space group**	P-1	P-1	P-1
**a/ Å**	8.8778(63)	8.6484(39)	8.7784(63)
**b/ Å**	12.109(3)	11.977(2)	11.991(35)
**c/ Å**	20.143(11)	39.183(16)	19.026(15)
**α/ °**	81.328(45)	86.932(27)	79.401(46)
**β/ °**	82.689(47)	85.509(30)	81.534(48)
**γ/ °**	74.466(34)	73.683(17)	75.300(35)
**R_{wp}**	7.084	2.3516	1.3664
**Phase fraction[a]**	98%	92%	81%

[a]The representative error of the phase fractions cannot be calculated because of the graininess of the sample.

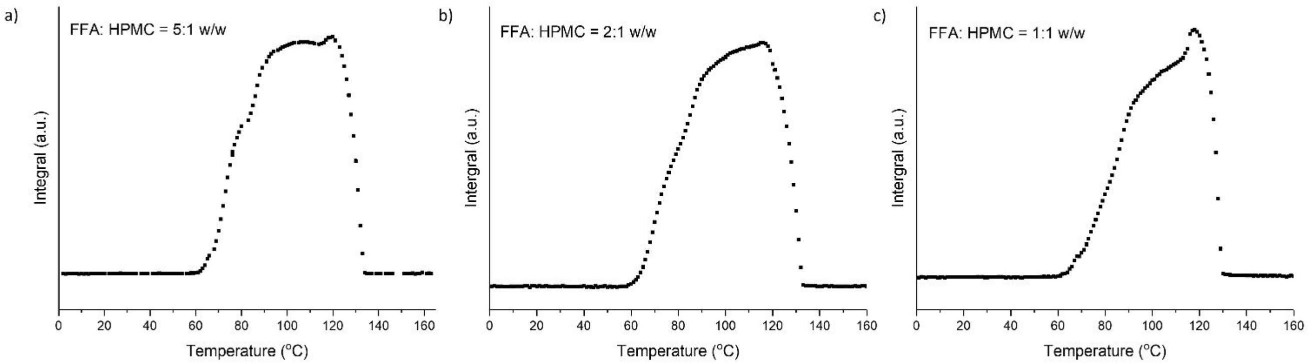

Figure S25. Plot of integrated total diffracted intensity for FFA form IV as a function of temperature with FFA: HPMC 100000 cp composites.

S10. 1:5 w/w FFA/ 100000 cp HPMC DISPERSION

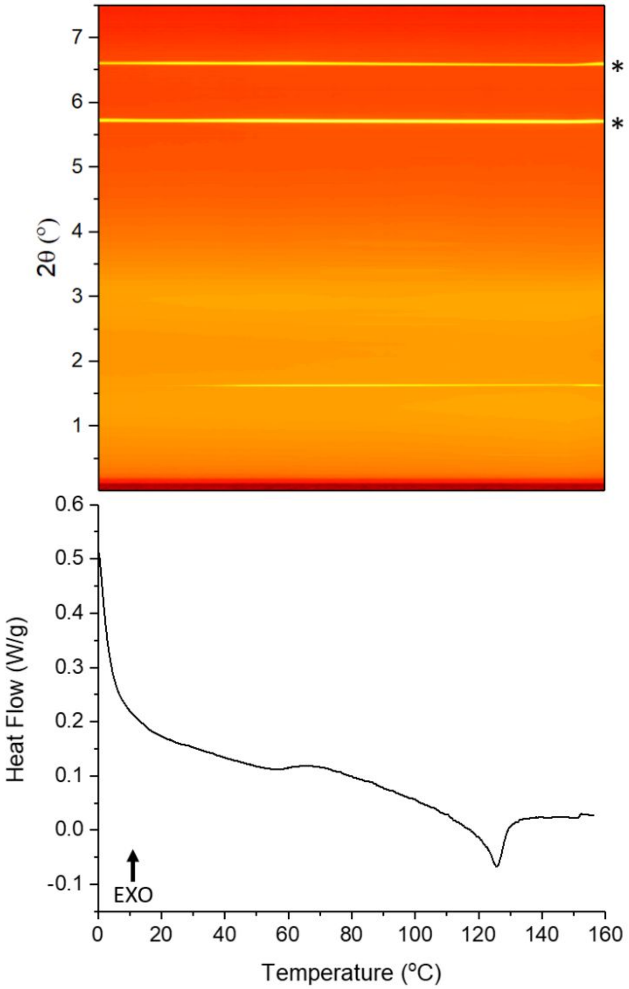

Figure S26. XRD-DSC data obtained during the reheating of a 1:5 w/w FFA/ HPMC 100000 cp ASD. Reflections marked * are a result of the aluminium pan.

S11. 5:1 w/w 4 cp FFA/ EC DISPERSIONS

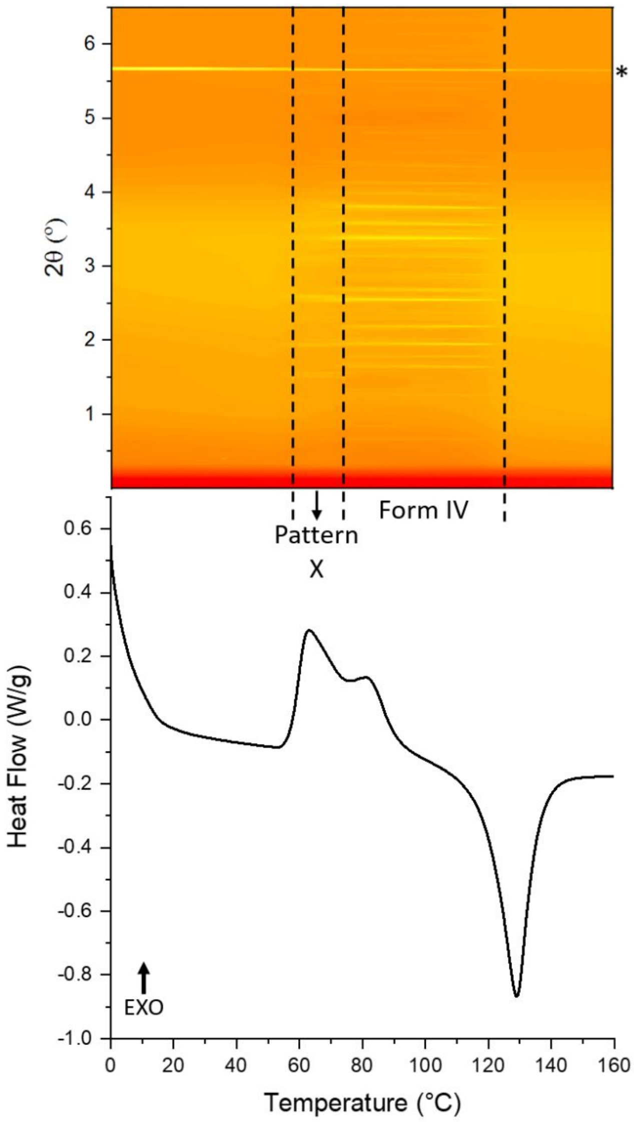

Figure S27. XRD-DSC data for the reheating of a 5:1 w/w FFA/EC ASD. Reflections marked * are a result of the aluminium pan.

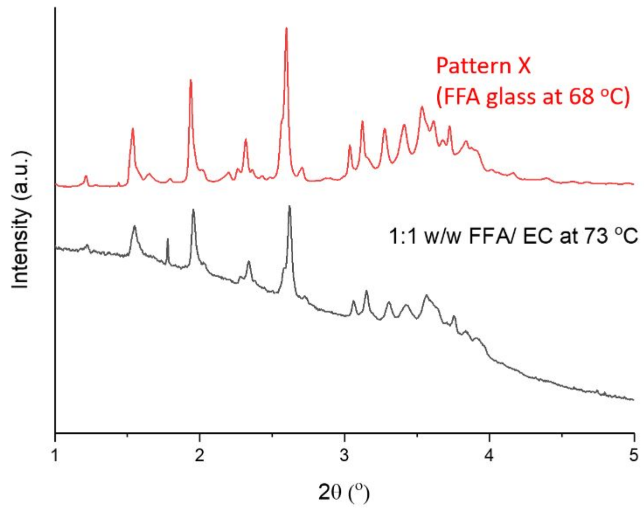

Figure S28. The observed XRD of form X generated from an FFA glass and that of a 5:1 w/w FFA/EC ASD at 73 ^{o}C during reheating.

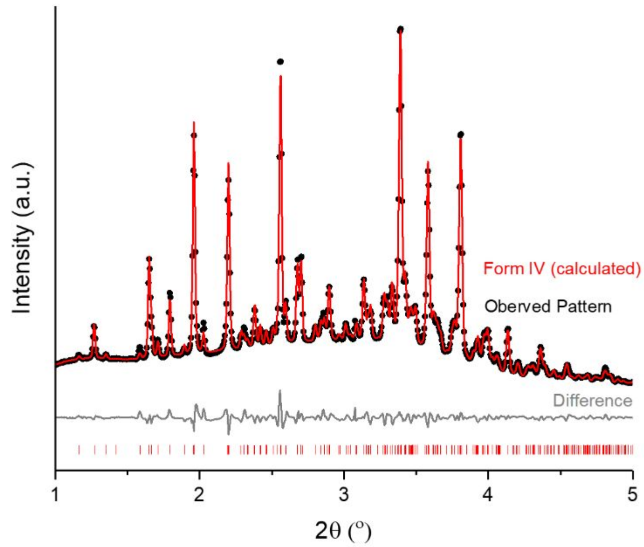

Figure S29. Rietveld refinement for a diffraction pattern recorded at 110 ^{o}C during reheating the 5:1 w/w FFA/EC ASD. Tick marks show the position of allowed reflections of FFA Form IV (FPAMCA15).

Table S9. Refinement parameters for the 5:1 w/w FFA/EC ASD at 110 ^{o}C. The starting model was taken from the CSD (form IV: FPAMCA15).

**Form**	**IV**
**Temperature (^{o}C)**	98
**Space group**	P-1
**a/ Å**	8.8530(13)
**b/ Å**	12.697(1)
**c/ Å**	20.259(2)
**α/ ^{o}**	80.493(7)
**β/ ^{o}**	81.044(6)
**γ/ ^{o}**	74.032(6)
**R_{wp}**	2.9997
**Phase fraction[a]**	/

[a]The phase fraction cannot be calculated because there is no reference for form X.

S12. 2:1 and 1:1 w/w FFA/ EC DISPERSIONS

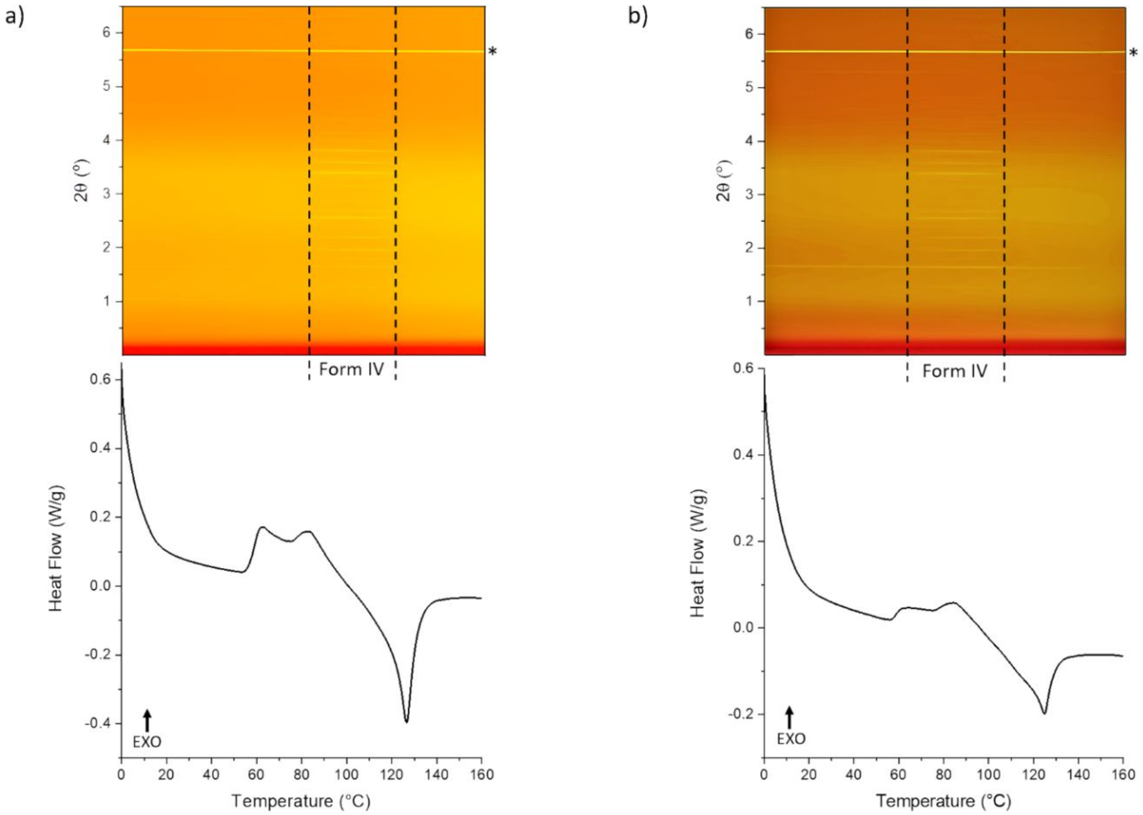

Figure S30. XRD-DSC data for the reheating of FFA/EC ASDs at a) 2:1 w/w; b) 1:1 w/w. Reflections marked * are a result of the aluminium pan.

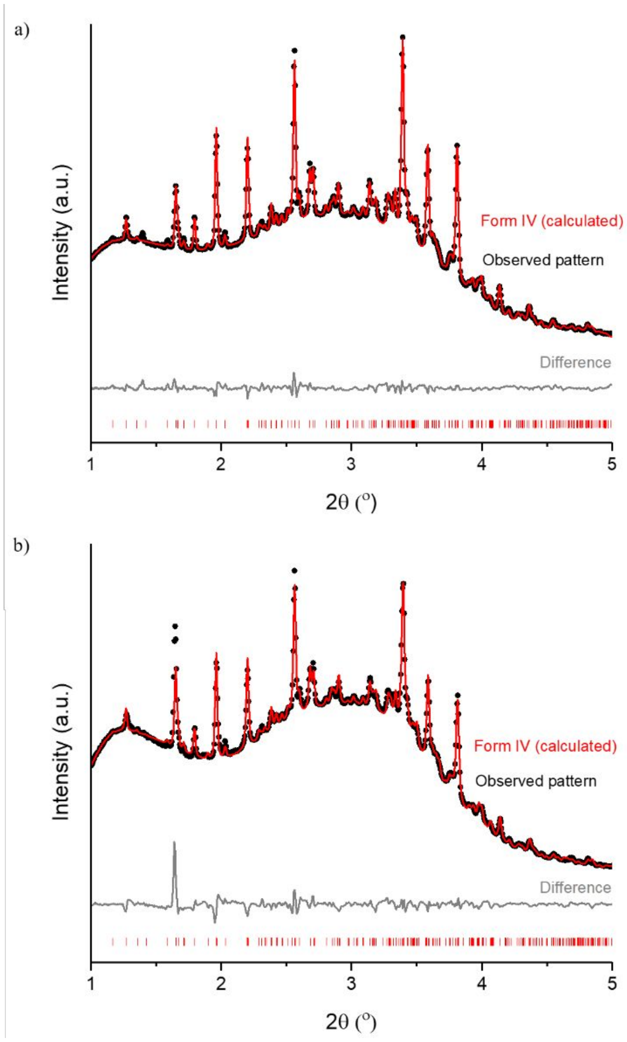

Figure S31. Rietveld refinement for diffraction patterns recorded during reheating FFA/EC ASDs. a) 2:1 w/w at 112 ^{o}C; b) 1:1 w/w at 111 ^{o}C. Tick marks show the position of allowed reflections of FFA form IV (FPAMCA15).

Table S10. Refinement parameters for 2:1 and 1:1 w/w FFA/EC systems. The starting model was taken from the CSD (form IV: FPAMCA15).

Form	IV (2:1 w/w)	IV (1:1 w/w)
**Temperature (°C)**	112	111
**Space group**	P-1	P-1
**a/ Å**	8.8473(17)	8.7784(29)
**b/ Å**	12.060(1)	11.988(2)
**c/ Å**	20.244(3)	20.001(6)
**α/ °**	80.475(9)	81.280(21)
**β/ °**	81.0420(8)	82.387(21)
**γ/ °**	74.031(7)	74.406(19)
**R_{wp}**	1.2484	2.0671
**Phase fraction[a]**	79%	89%

[a]The representative error of the phase fractions cannot be calculated because of the graininess of the sample.

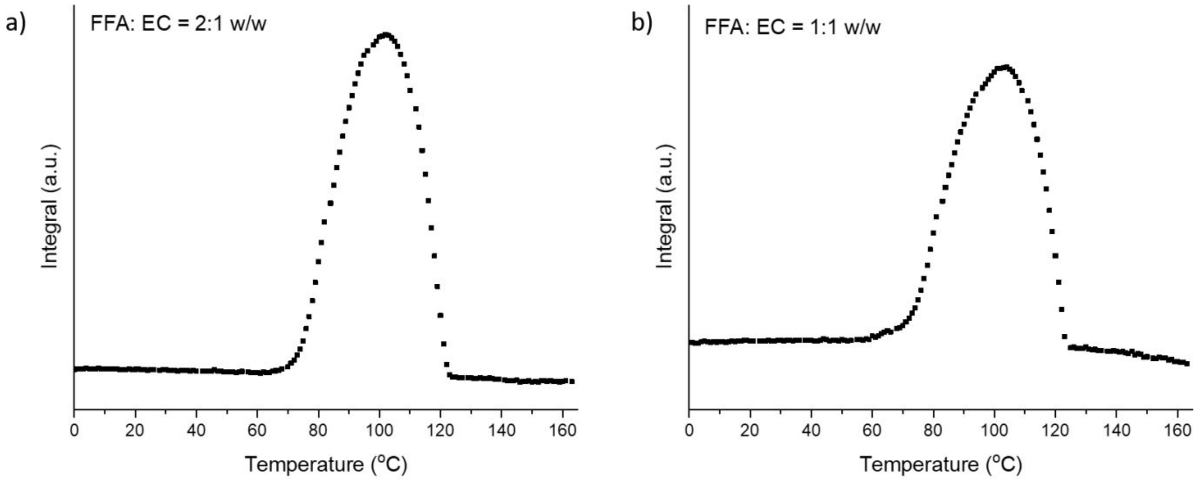

Figure S32. Plot of integrated total diffracted intensity for FFA form IV as a function of temperature with a) 2:1 w/w, b) 1:1 w/w FFA/ EC composites.

S13. 1:5 w/w FFA/ EC DISPERSION

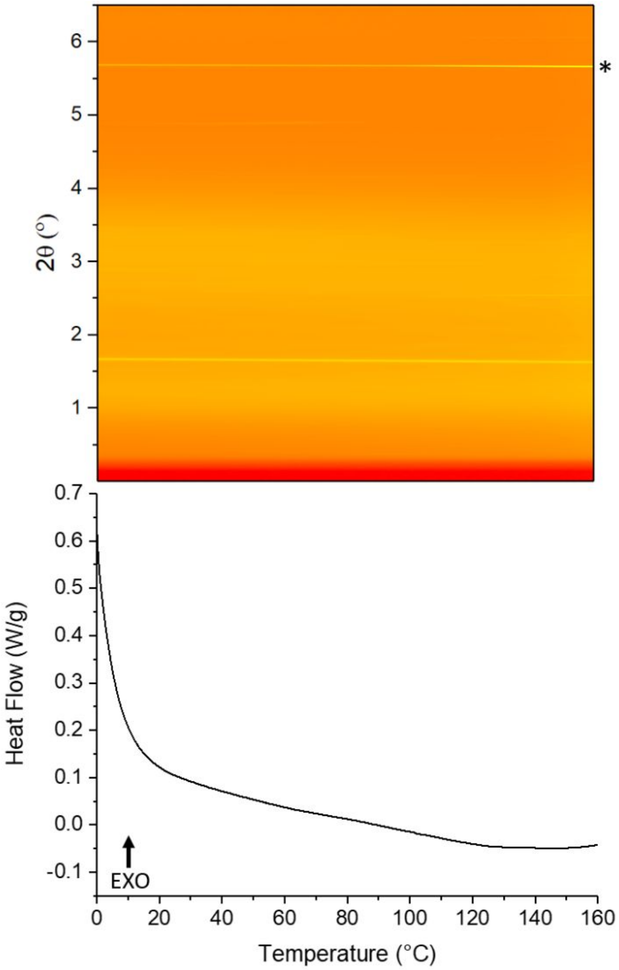

Figure S33. XRD-DSC data for the reheating of a 1:5 w/w FFA/EC ASD. Reflections marked * are a result of the aluminium pan.

REFERENCES

1. Case, D. H.; Srirambhatla, V. K.; Guo, R.; Watson, R. E.; Price, L. S.; Polyzois, H.; Cockcroft, J. K.; Florence, A. J.; Tocher, D. A.; Price, S. L., Successful Computationally Directed Templating of Metastable Pharmaceutical Polymorphs. *Cryst. Growth Des.* **2018,** *18* (9), 5322-5331.

2. López-Mejías, V.; Kampf, J. W.; Matzger, A. J., Nonamorphism in Flufenamic Acid and a New Record for a Polymorphic Compound with Solved Structures. *J. Am. Chem. Soc.* **2012,** *134* (24), 9872-9875.
